# Ticks without borders: microbiome of immature neotropical tick species parasitizing migratory songbirds along northern Gulf of Mexico

**DOI:** 10.3389/fcimb.2024.1472598

**Published:** 2024-11-18

**Authors:** Shahid Karim, Theodore J. Zenzal, Lorenza Beati, Raima Sen, Abdulsalam Adegoke, Deepak Kumar, Latoyia P. Downs, Mario Keko, Ashly Nussbaum, Daniel J. Becker, Frank R. Moore

**Affiliations:** ^1^ School of Biological, Environmental, and Earth Sciences, University of Southern Mississippi, Hattiesburg, MS, United States; ^2^ United States Geological Survey, Wetland and Aquatic Research Center, Lafayette, LA, United States; ^3^ Institute for Coastal Plain Science, Georgia Southern University, Statesboro, GA, United States; ^4^ School of Biological Sciences, University of Oklahoma, Norman, OK, United States

**Keywords:** migratory birds, exotic species, tick prevalence, 16s sequencing, microbiome, endosymbiont

## Abstract

**Introduction:**

The long-distance, seasonal migrations of birds make them an effective ecological bridge for the movement of ticks. The introduction of exotic tick species to new geographical regions can cause the emergence of novel tick-borne pathogens. This study examined the prevalence of exotic tick species parasitizing migratory songbirds at stopover sites along the northern Gulf of Mexico using the mitochondrial 12S rRNA gene.

**Methods:**

Overall, 421 individual ticks in the genera *Amblyomma*, *Haemaphysalis*, and *Ixodes* were recorded from 28 songbird species, of which *Amblyomma* and *Amblyomma longirostre* were the most abundant tick genera and species, respectively. A high throughput 16S ribosomal RNA sequencing approach characterized the microbial communities and identified pathogenic microbes in all tick samples.

**Results and discussion:**

Microbial profiles showed that Proteobacteria was the most abundant phylum. The most abundant pathogens were *Rickettsia* and endosymbiont *Francisella*, *Candidatus Midichloria*, and *Spiroplasma*. Permutation multivariate analysis of variance revealed that the relative abundance of *Francisella* and *Rickettsia* drives microbial patterns across the tick genera. We also noted a higher percentage of positive correlations in microbe-microbe interactions among members of the microbial communities. Network analysis suggested a negative correlation between a) *Francisella* and *Rickettsia* and, b) *Francisella* and *Cutibacterium*. Lastly, mapping the distributions of bird species parasitized during spring migrations highlighted geographic hotspots where migratory songbirds could disperse ticks and their pathogens at stopover sites or upon arrival to their breeding grounds, the latter showing mean dispersal distances from 421–5003 kilometers. These findings spotlight the potential role of migratory birds in the epidemiology of tick-borne pathogens.

## Graphical Abstract

Overview of the experimental approach for bird collection and characterization of neotropical ticks microbiome. **(A)** Birds migrating through the Mississippi flyway into the United States were trapped and captured for identification and tick collection and **(B)** immature developmental stages of feeding ticks infesting the birds were carefully removed and stored for morphological and molecular identification of tick species. **(C)** Tick DNA was used for the PCR amplification of arthropods mitochondrial 12S rRNA and amplicons were sequenced and compared to deposited datasets in NCBI database. **(D)** Microbial DNA was isolated using V3-V4 16S primers and unique barcodes attached to amplified 16S sequences prior to Illumina MiSeq sequences. **(E)** Distribution and contribution of birds to tick infestation, and distribution and community profiles of sequenced microbial communities.

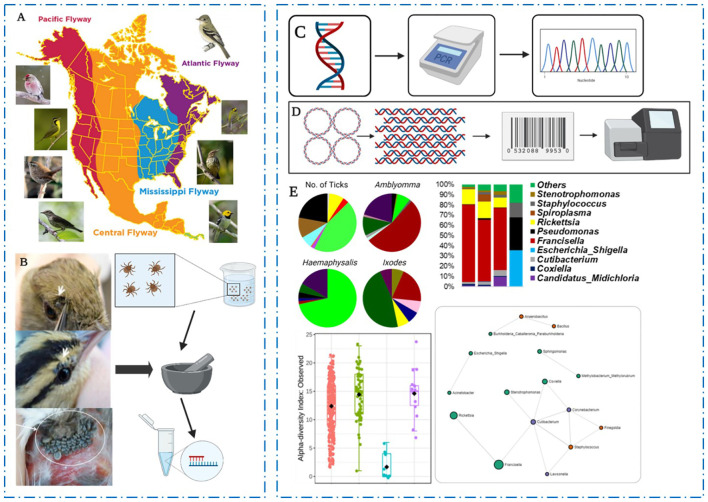

## Introduction

Ixodid (hard-bodied) ticks have a broad host range and are globally distributed, facilitating the transmission of bacterial, viral, and protozoan pathogens that cause diseases in humans and animals (Sonenshine, 1993). Transmission of tick-borne pathogens to humans places a significant impact on both public health and the economy, including substantial costs of treatment and disability associated with post-recovery states (Rochlin and Toledo, 2020). In North America, ticks are responsible for over 95% of vector-borne diseases (Rosenberg et al., 2018).

The most prevalent tick-borne illness is Lyme borreliosis, which is caused by the spirochete *Borrelia burgdorferi* sensu lato complex and vectored by *Ixodes scapularis*. While *I. scapularis* is the primary vector for *B. burgdorferi*, *I. pacificus* also plays a significant role as a vector in the western United States. Known as the western black-legged tick, *I. pacificus* is primarily found in the western regions of the United States and Canada, with the highest prevalence in California, though it is also present in Oregon, Washington, Utah, Nevada, and Arizona. Over 300,000 and 85,000 cases are reported annually in the USA and Europe, respectively (Lindgren and Jaenson, 2006). Other tick-borne bacterial pathogens found in the USA include but are not limited to *Rickettsia rickettsii, Anaplasma phagocytophilum, R. parkeri, Ehrlichia chaffeensis*, and *E. ewingii*. The emergence of the invasive Asian long-horned tick, *Haemaphysalis longicornis*, in the USA (Rainey et al., 2018; Rochlin, 2019) poses a potential threat to the livestock industry, as it is the competent vector of *Theileria orientalis*, the protozoan that causes theileriosis (Fujisaki et al., 1994; Hammer et al., 2015), which is characterized by anemia and occasional mortality.

Although terrestrial mammals commonly serve as hosts for ticks, they typically travel relatively short distances before ticks complete their blood meal and drop off (Cheng, 1969). However, ticks of the genera *Argas, Ornithodoros, Ixodes, Amblyomma*, and *Haemaphysalis* also can parasitize birds (James et al., 2011; Barros-Battesti et al., 2006; Hasle et al., 2011; Movila et al., 2011; Zeringóta et al., 2017). In the case of migratory avian hosts, ticks can disperse much further from their origin. Many species of migratory songbirds, for example, fly long distances, sometimes moving across continents and extensive geographical features (e.g., oceans, deserts) within a relatively short period every spring and autumn (Deppe et al., 2015; Ouwehand and Both, 2016). Due to the energetic cost of migration (Wikelski et al., 2003; McWilliams et al., 2004), most migratory birds must stop *en route* at stopover sites to rest and restore their energy reserves in unfamiliar habitats. Birds may pick up new or drop off existing ectoparasites, such as ticks, during stopover, acting as a long-distance dispersal mechanism for ectoparasites and their pathogens (Mukherjee et al., 2014; Cohen et al., 2015; Zenzal et al., 2017; James et al., 2011; Hasle, 2013; Nicholls and Callister, 1996; Becker et al., 2022). Moreover, migratory birds often serve as competent reservoirs of multiple tick-borne pathogens (Reed et al., 2003; Comstedt et al., 2006; Becker and Han, 2021) and thus are rightfully termed as “ecological bridges” due to their role in moving both ectoparasites and pathogens to new ecological niches (Pascucci et al., 2019).

Exotic ticks transported into North America by migratory birds have thus far had limited success in becoming locally established, which may stem from the unavailability of suitable climatic conditions and/or natural host species as well as competition with native ticks (Hasle, 2013). If ticks transported by migratory birds were to succeed in establishing permanent populations in North America, the pathogens they carry may start infecting local native hosts and tick species. With a changing climate, non-native tick species dispersed by migratory birds could eventually establish populations in the USA with the potential of becoming invasive.

Invasive species cost over $26 billion per year in the USA in economic, environmental, and public health damages (Crystal-Ornelas et al., 2021). One example is the recent invasion of *Haemaphysalis longicornis*, the Asian long-horned tick. This invasive species was first identified in New Jersey in 2017 (Rainey et al., 2018) and was then confirmed in at least 14 other states (USDA APHIS, 2021; Tufts et al., 2019). *H. longicornis* is native to East Asia, and geographic expansion could present a significant threat to animal and public health by serving as a competent vector of pathogens including *Theileria, Babesia, Rickettsia*, and severe fever with thrombocytopenia syndrome virus (Thompson et al., 2020; Luo et al., 2015; Fill et al., 2017; Godsey et al., 2016). Additionally, *H. longicornis* is able to reproduce parthenogenetically (without a male), furthering the potential spread and impact of the species.

This study aimed to investigate the diversity of commensal and pathogenic bacteria within the microbial community of Neotropical ticks collected from songbirds during spring and autumn when many of them stopover along the northern Gulf of Mexico coast when entering or leaving the USA. We wanted to determine if ticks infesting birds can serve as sentinels to monitor the introduction of known and previously unreported pathogenic bacteria into new geographical areas. Lastly, we assessed geographic overlap in the migration and breeding distributions of songbirds found to be carrying ticks during spring migration to identify spatial hotspots of potential tick and pathogen dispersal capacity into the USA.

## Materials and methods

### Ethics approval

All animal sampling was conducted in strict accordance with the recommendations in the Guide for Care and Use of Laboratory Animals of the National Institutes of Health, USA. Collection and handling of birds were approved by the U.S. Geological Survey Bird Banding Laboratory (permit #24101), the Louisiana Department of Wildlife and Fisheries, the Alabama Department of Conservation and Natural Resources, and the Institutional Animal Care and Use Committee (IACUC) at the University of Southern Mississippi (protocol #17081101) and the U.S. Geological Survey (protocol #LFT 2019-05).

### Bird capture and sampling

Songbirds were sampled at six stopover sites along the northern Gulf of Mexico during autumn (late August to early November) in 2018 and 2019 and seven stopover sites during spring (mid-March to mid-May) in 2018 and 2019. Birds were captured using 15–20 nylon mist nets (12 or 6 x 2.6 m; 30-mm mesh) per site from up to four sites in southwest Louisiana and three sites in southern Alabama (**Table 1**). Netting was conducted daily with the exception of inclement weather. During spring, nets were operated from local sunrise until approximately five hours after sunrise; during three days of the week, nets were reopened from three hours before local sunset until ~30 minutes before sunset. During autumn, nets were operated from local sunrise until approximately six hours after sunrise. Each captured individual was ringed with a uniquely numbered U.S. Geological Survey metal leg band, had its condition assessed, morphometrics recorded, and, when time allowed, inspected for the presence of ectoparasites. Our classification of bird species included three categories: resident, short-distance migrants, and long-distance migrants, primarily determined by their predominant location during the non-breeding period. Resident species were present throughout the year at our study sites. Short-distance migratory species were observed spending the non-breeding season primarily north of the Tropic of Cancer, while long-distance migratory species predominantly inhabited regions south of the Tropic of Cancer during the stationary non-breeding period (DeGraaf and Rappole, 1995; Carlisle et al., 2004; Zenzal et al., 2018).

**Table 1:** Bird trapping and tick collection sites including state and coordinates.

Site	State	Coordinates
*Grand Chenier*	Louisiana (LA)	29.749659, -92.894811
*Grand Chenier*	Louisiana (LA)	29.738521, -92.836370
*Hayes*	Louisiana (LA)	0.109666, -92.908688
*Reeves*	Louisiana (LA)	30.435878, -93.075420
*Gulf Shores*	Alabama (AL)	30.260475, -87.748360
*Saraland*	Alabama (AL)	30.811519, -88.058210
*Perdido*	Alabama (AL)	31.024289, -87.677632

### Tick collection and molecular identification

When a tick was discovered attached to a bird, we collected the tick in the field by carefully detaching it with fine-tipped forceps and preserved it in a vial of 70% ethanol. For each tick sample, we recorded the collection date, unique sample code, and the bird’s unique leg band number. Ticks collected from songbirds were mostly immature developmental stages. Taxonomic keys for immature stages of exotic or uncommon ticks are not available for all species. Furthermore, engorged immature specimens are often damaged to such an extent that diagnostic morphological characters are obliterated. Therefore, individual whole ticks were molecularly identified by using 12S DNA sequencing. A fragment of each tick’s mitochondrial 12S rRNA gene (~360 bp) sequence was amplified from 485 samples with T1B and T2A primers (Beati and Keirans, 2001) obtained from Invitrogen. Dream Taq Polymerase (Invitrogen, Thermo Fisher Scientific, Waltham, MA, USA) was used to amplify tick DNA. The amplicons were bidirectionally sequenced at Eurofins Genomics (Eurofins Genomics, Louisville, KY, USA). Complementary strands were visually examined and assembled by using Benchling. The sequences were compared to homologous nucleotide fragments in GenBank using BLAST (Mukherjee et al., 2014). Sequencing data was submitted to GenBank and the accession number for individual ticks was provided (**Supplementary Table S1**).

### Statistical analysis of tick infestation

To analyze tick infestation data, we used generalized linear mixed models (GLMMs) via the *lme4* package in the R statistical language (Bates et al., 2015; R Core Team 2022). We used a two-step process to quantify species-level variation in tick outcomes and to assess additional temporal and migratory drivers of infestation. For binary tick positivity across all birds sampled during our study (*n* = 17,550), we first fit a GLMM with a fixed effect of bird species and a site-level random effect to account for regional artifacts. We then had another GLMM with an additional random effect for bird species that included fixed effects of year, migratory season, and their interaction. To analyze the intensity of tick parasitism on birds with identified ticks (*n* = 164), we likewise fit a GLMM with Poisson errors and an observation-level random effect nested within the site random effect to account for overdispersion (Harrison, 2014). We then fit another equivalent GLMM with an additional random effect for bird species that included fixed effects of year, migratory season, and migratory category. We excluded bird species with only one tick association (*n* = 7 species) from these intensity GLMMs. We assessed Poisson GLMMs for overdispersion using the *performance* package and adjusted any *post-hoc* comparisons from our models using the Benjamini–Hochberg correction. We derived model fit with marginal and condition R^2^ via the *MuMIn* package (Nakagawa and Schielzeth, 2013). Statistical significance of fixed effects from GLMMs were tested using a Type II analysis-of-variance (i.e., Wald *χ^2^* statistics) with the *car* package.

### Library preparation for illumina 16S sequencing

Sixty-two of the 421 genomic DNA isolated from individual ticks did not pass quality control (QC) and the 359 that passed QC were used to generate libraries according to the library generation protocol by Illumina Indexing Methodology (Kumar et al., 2022). Briefly, a two-stage PCR amplification process was used to amplify the 16S rRNA V3-V4 region, followed by a dual indexing step that assigns unique index sequences to the V3-V4 amplicons. The concentration of the resulting PCR products were determined using qPCR and equal concentration of each sample was pooled and sequenced in a single run of an Illumina MiSeq sequencing instrument using reagent kit v2 (500 cycles) with 2 X 250 bp output at the University of Mississippi Medical Centre (UMMC) Genomics Core Facility. DNA extraction controls, PCR controls, and known mock bacterial communities (ZymoBIOMICS™ Microbial Community DNA Standard, Irvine, CA, USA) were simultaneously processed alongside the tick samples. All critical steps including determination of amplicon size, and amplicon purification following each PCR step were performed as described by Kumar et al. (2022).

### 16S sequence analysis

Unless otherwise stated, all data preprocessing was done following the video tutorial of the Quantitative Insights into Microbial Ecology (QIIME2, v2022.2) pipeline (Bolyen et al., 2019). Briefly, demultiplexed fastq files were unzipped and the forward and reverse fastq files merged into a single fastq file using Casava (v1.8). The Atacama soil microbiome pipeline was incorporated to control demultiplexed paired-end reads using the DADA2 plugin as previously described (Callahan et al., 2016). Low-quality and Chimeric sequences were trimmed; subsequent merging of paired-end reads ensured 20 nucleotide overhangs between forward and reverse reads and the chimeric seqeuences removed from the sequence table. The non-chimeric representative sequences were aligned and construction of phylogenetic tree performed using MAFFT v. 7 and FasTree v. 2.1 plugins. Operational taxonomic assignment was performed using the qiime2 feature-classifier plugin v. 7.0, which was previously trained against the SILVA 138.1 database preclustered at 99% (Quast et al., 2013). Tables representing operational taxonomic units (OTUs) and representative taxonomy were exported and used for diversity metric analysis using the Microbiome Analyst web-based interface (Dhariwal et al., 2017; Chong et al., 2020). Raw sequences were submitted to the GenBank (BioProject # PRJNA1047655).

### Tick microbiome visualization

Microbiome Analyst, a web-based interface, was used for data visualization by employing taxonomy and metadata tables generated from data processing as input files (Dhariwal et al., 2017; Chong et al., 2020). Low count and prevalence data were filtered from the OTU table by setting values of 10 and 20, respectively. A filtered abundance table was exported and used in generating histograms of bacterial abundance in Microsoft Excel 2016 (Microsoft, 2018). Network correlation maps were constructed based on the sparse correlations for compositional data (SparCC) approach (Friedman and Alm, 2012). This approach uses the log-transformed values to carry out multiple iterations, which subsequently identifies taxa outliers to the correlation parameters (Chong et al., 2020). To compare the differences in the microbiome between tick groups based on measures of distance or dissimilarity, Bray-Curtis dissimilarity was used to calculate the differences in feature abundance and ordination of the plots was visualized using Principal Coordinates Analysis (PCoA).

### Statistical analysis of tick microbial communities

The statistical analysis was carried out in Microbiome Analyst (Dhariwal et al., 2017; Chong et al., 2020). Kruskal–Wallis tests followed by Dunn’s multiple comparison tests were used to compare the differences in alpha diversity between all identified ticks at the genus and species level based on the observed OTU metric and Shannon’s diversity index. Permutational multivariate analysis of variance (PERMANOVA) was used to determine significant pairwise differences in the tick microbial communities by comparing the means across different tick genus and species. Statistically significant data were represented as P <0.05.

### Spatial analysis of parasitized birds

To identify spatial hotspots of potential tick and pathogen dispersal into North America, we mapped the distributions of bird species parasitized by ticks during spring migration, when detected ticks most likely originated from Central and South American non-breeding grounds. For those parasitized migratory birds, we aggregated species shapefiles from the International Union for Conservation of Nature using the *rgdal* and *rgeos* packages in the R statistical language (Baillie et al., 2004). Aggregated avian distributions were stratified by migration and breeding seasons to illustrate dispersal capacity to stopover sites and the breeding grounds respectively, for each identified tick species (Baillie et al., 2004). Lastly, we calculated distance between spring capture site and the centroid of the breeding range for each parasitized bird species using the *geosphere* package, for each unique combination of site, bird species, and tick species. We then used a GLM with a Gamma distribution to identify how mean dispersal capacity during spring migration varied across bird-infesting tick species, again using Type II analysis-of-variance to assess statistical significance.

## Results

### Tick infestation and identification

A total of 164 individual birds of 28 species were found with attached ticks during our spring and autumn sampling periods (**Supplementary Table S1**). When considering tick parasitism status across the 17,550 birds sampled, bird species did not significantly differ in their odds of infestation (χ^2^ = 104.85, df = 103, P = 0.43, R^2^_m_ = R^2^_c_ = 0.22), likely owing to the overall low prevalence of parasitism (<1%). After accounting for site and species variation, tick positivity varied significantly by sampling year and migratory season (interaction: χ^2^ = 5.96, P = 0.01, R^2^_m_ = 0.19, R^2^_c_ = 0.20). The odds of birds harboring ticks were greatest in spring 2019 compared to all other sampling periods (*z* > 5.2, P < 0.01).

**Figure 1:**
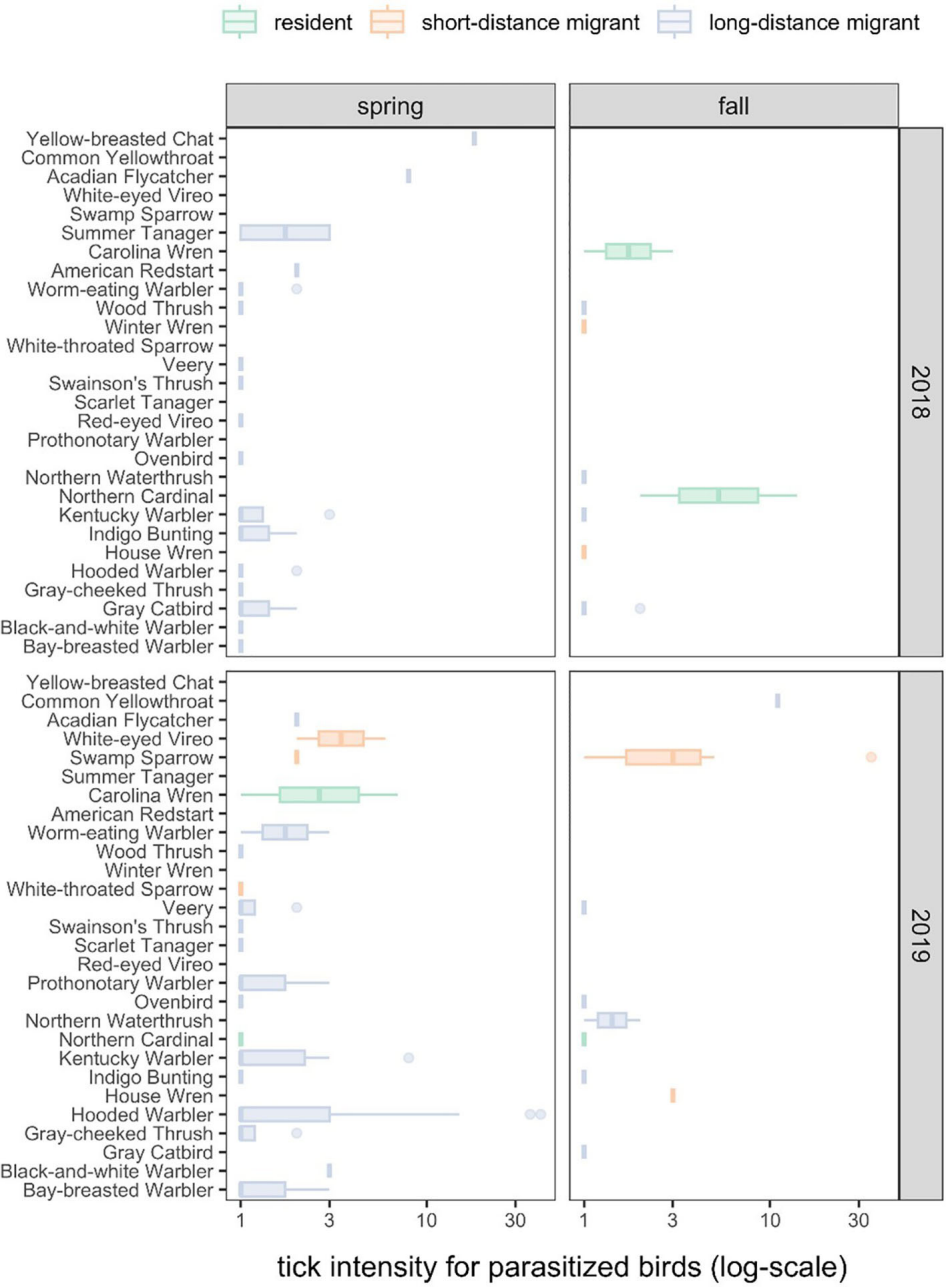
The distribution of tick intensities across bird species, years, and seasons.

Tick intensities among parasitized birds were highly variable across host species (**Figure 1**; χ^2^ = 33.93, df = 20, P = 0.03), which explained 17% of the variance (R^2^_m_) compared to the individual- and site-level random effects (R^2^_c_ = 0.54). The Yellow-breasted Chat (*Icteria virens*) and Common Yellowthroat (*Geothlypis trichas*) had the greatest tick intensities (x̄ = 18 and 11), although only one individual per species was parasitized. Most ticks were contributed by the Hooded Warber (*Setophaga citrina*, 31%) and Swamp Sparrow (*Melospiza georgiana*, 16%), whereas the least ticks were contributed by the Red-eyed Vireo (*Vireo olivaceus*, <1%), Scarlet Tanager (*Piranga olivacea*, <1%), White-throated Sparrow (*Zonotrichia albicollis*, <1%), Winter Wren (*Troglodytes hiemalis*,<1%), American Redstart (*Setophaga ruticilla*, <1%), and Northern Waterthrush (*Parkesia noveboracensis*, <1%). Most ticks (54%) were collected at the stopover sites in Grand Chenier, LA.

Our secondary Poisson GLMM (R^2^_m_ = 0.08, R^2^_c_ = 0.52) revealed no significant variation in tick intensity by migratory season (χ^2^ = 0.28, P = 0.60) or year (χ^2^ = 2.38, P = 0.12), although birds tended to have more ticks in 2019 (x̄ = 3.09) and during autumn migration (x̄ = 3.00). Tick intensities did vary by migration category (χ^2^ = 7.24, P = 0.03), which was driven by short-distance migrants having greater tick intensities on average than long-distance migrants (x̄ = 4.15 vs x̄ = 2.24; z = 2.36, P = 0.05), neither of which differed significantly from residents (x̄ = 3.44; short-distance migrants: z = 0.22, P = 0.82; long-distance migrants: z = 1.82, P = 0.10). Because short- and long-distance migratory birds were more commonly sampled, these species contributed 73% and 20% of all identified ticks (**Figure 1**). Most ticks were collected in 2019 (71%) and during spring migration (70%). Neither of our tick intensity GLMMs showed significant overdispersion (χ^2^ ≥ 18.74, P = 1).

Of the 421 tick samples collected from songbirds, 62 did not meet quality control standards for amplification and sequencing. The remaining 359 specimens were identified as *Amblyomma americanum, A. maculatum, A. calcaratum, A. coelebs, A. geayi, A. longirostre, A. nodosum, A. ovale, A. parvum, A. sabanerae, A. triste, A. varium, Haemaphysalis leporispalustris, Ixodes brunneus, I. dentatus*, and *I. scapularis*. Four tick species accounted for 81% of all ticks collected during the study period, with *A. nodosum* (29%; 120/421), *A. longirostre* (20%; 83/421)*, H. leporispalustris* (16%; 66/421), and *A. maculatum* (16%; 69/421) representing the most tick species, respectively (**Supplementary Table S1**).

### Tick DNA sequencing results

Illumina 16S rRNA sequencing of all 359 tick DNA samples produced 11,064,738 raw forward and reverse reads. The distribution of the raw reads ranges from an average of 26,957 minimum reads per sample and a maximum of 89,223 reads per sample (**Supplementary Table S2**). Rarefaction analysis of the individual samples from a sequencing depth of 500 to 5,000 raw reads confirmed there was adequate sequence coverage relative to the number of observed features and a plateau for individual tick samples (**Supplementary Figure S1**).

### Phylum-level abundance and genus distribution in tick microbiota

**Figure 2:**
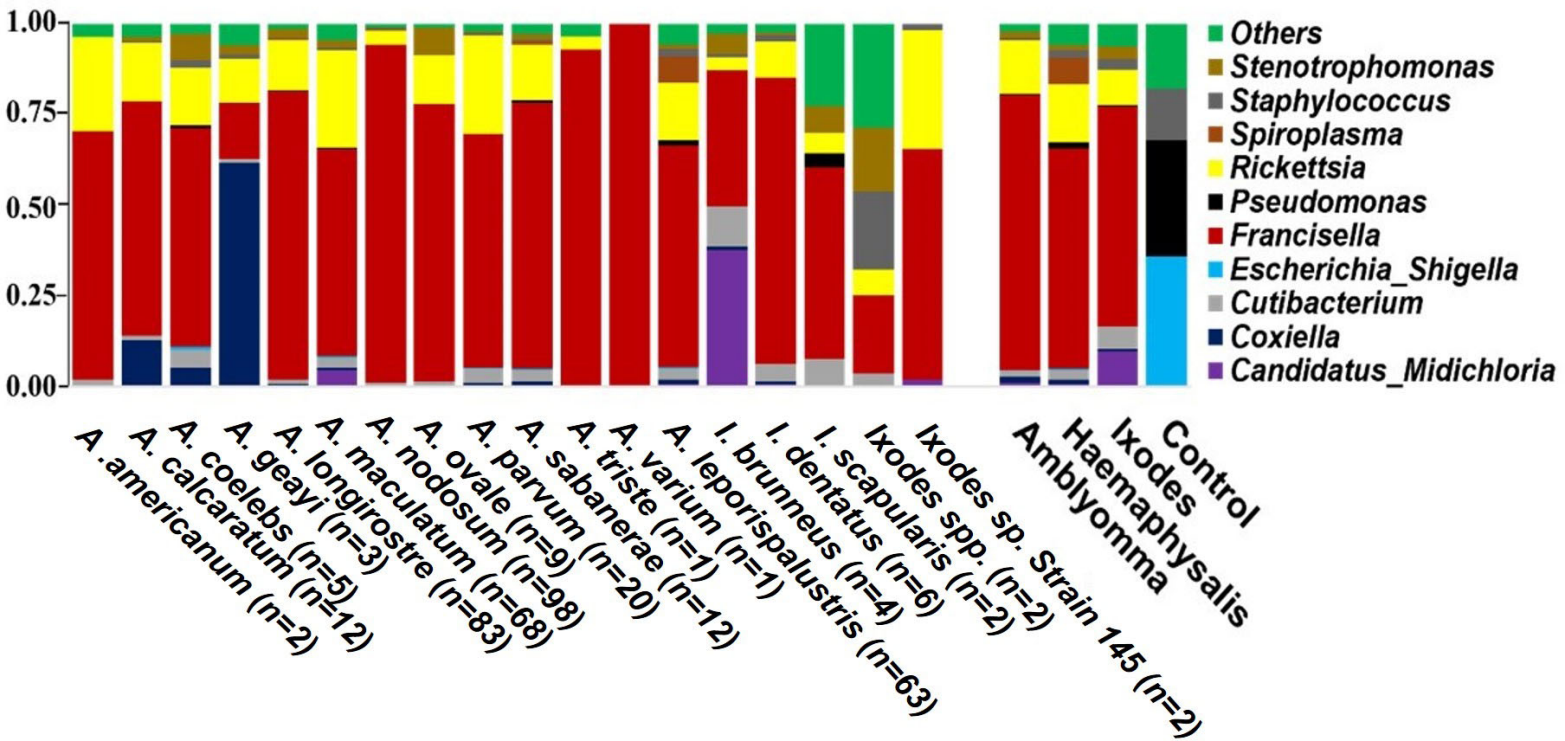
Relative abundance of the top 10 bacterial OTUs identified at the genus level. Each horizontal bar represents the cumulative average of the bacterial abundance identified from each tick species and genus. Mock bacterial communities from ZymoBIOMICS™ were included as control.

Overall, 1,416 unique bacterial OTUs were identified across each tick genera (**Supplementary Table S3**). Proteobacteria represented the most abundant phylum across the three genera of ticks sequenced (**Supplementary Table S4**). The abundance of the phylum Proteobacteria ranges from 82% to 98% of the total bacterial OTU (**Supplementary Figure S2A**; **Supplementary Table S4**). Other identified OTUs were either classified in the phyla Actinobacteriota, Firmicutes, Cyanobacteria, or unassigned to any known bacteria phyla. At the genus level, regardless of the tick species, *Francisella* accounted for the highest abundance of bacteria, ranging from 60% to 75%. Notably, *Francisella* was the sole bacteria genus identified in *A. varium* (**Figure 2**, **Supplementary Figure S2B**). Apart from *Francisella*, ten other bacteria genera had reads exceeding 1% in at least one tick (**Figure 2**, **Supplementary Figure S2B**). *Rickettsia* had the second highest relative abundance (11-14%) in all ticks, while *Spiroplasma* and *Cutibacterium* were present in relatively high abundance in *Haemaphysalis* and *Ixodes* ticks respectively (**Figure 2**; **Supplementary Figure S2B**). *Coxiella* was present in *A. coelebs* (5%), *A. calcaratum* (13%), and *A. geayi* (60%), while *Candidatus Midichloria* was present in *A. maculatum* (5%) *I. brunneus* (37%).

### Tick bacterial diversity

**Figure 3:**
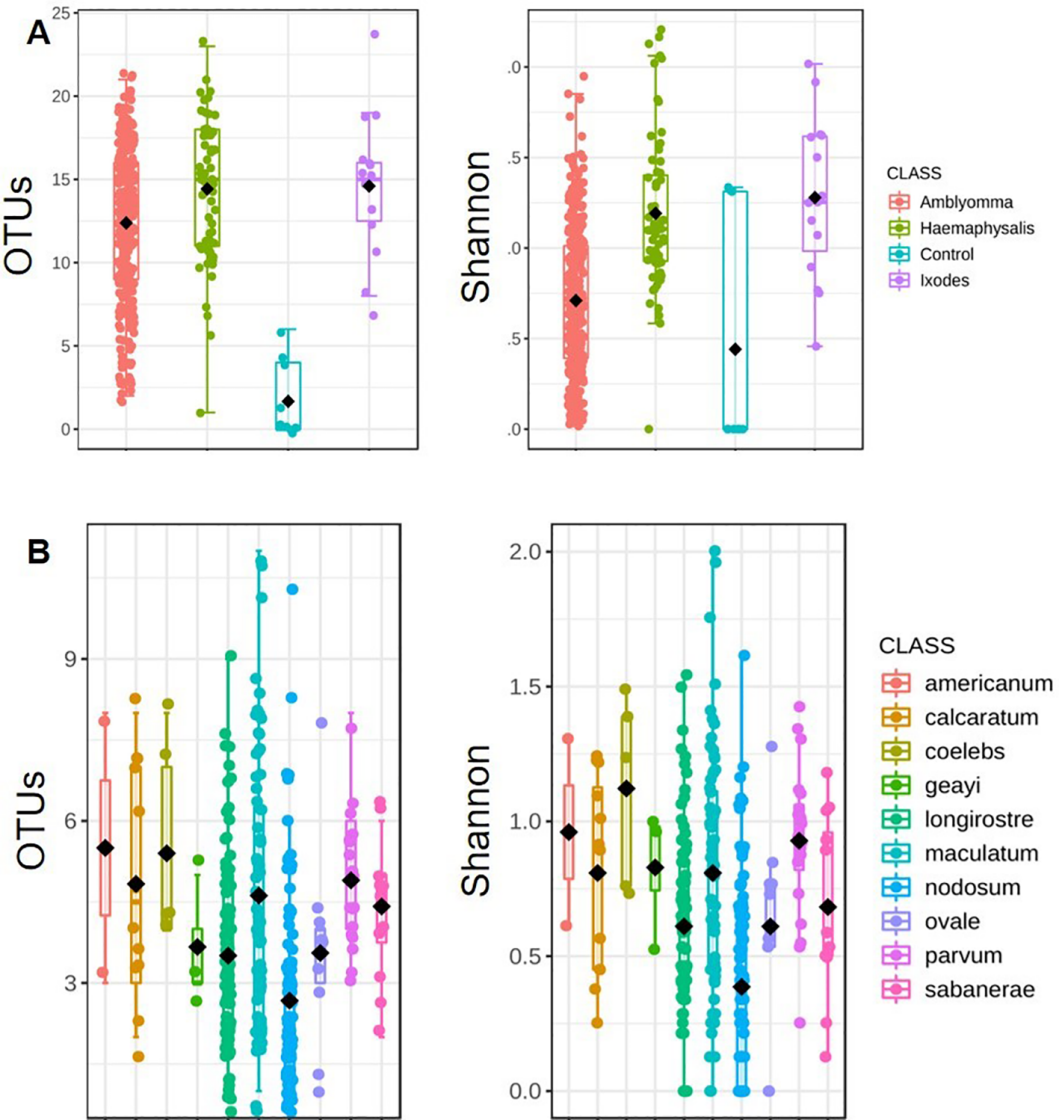
Measures of alpha diversity for **(A)** combined tick genera and **(B)** *Amblyomma* species. Alpha diversity was estimated using the observed OTUs and Shannon’s Index following rarefaction analysis of samples to a depth of 5000 sequence.

Ticks from the genus *Amblyomma* exhibited the lowest bacterial alpha diversity, whereas *Haemaphysalis* exhibited the highest alpha diversity (Observed OTUs: p-value: 9.6369e-13; [Kruskal-Wallis] statistic: 58.995; Shannon: p-value: 1.0478e-13; [Kruskal-Wallis] statistic: 63.504; **Figure 3A**). *A. coelebs* had the highest bacterial alpha diversity, while *A. nodosum* had the least diverse bacterial communities based on observed OTUs (p-value: 1.0466e-10; [Kruskal-Wallis] statistic: 65.716) and Shannon’s diversity index (p-value: 1.1177e-11; [Kruskal-Wallis] statistic: 70.685) when compared to all other *Amblyomma* species (**Figure 3B**). There was no significant difference in alpha diversity observed within the individual *H. leporispalustris* (p-value: 0.57981; [Kruskal-Wallis] statistic: 3.7915) and between *Ixodes* genera (Observed OTUs: p-value: 0.57037; [Kruskal-Wallis] statistic: 2.0098; Shannon’s Index: p-value: 0.28437; [Kruskal-Wallis] statistic: 3.7958; Simpson’s Index: p-value: 0.23367; [Kruskal-Wallis] statistic: 4.2708). (**Supplementary Figure S3**).

**Figure 4:**
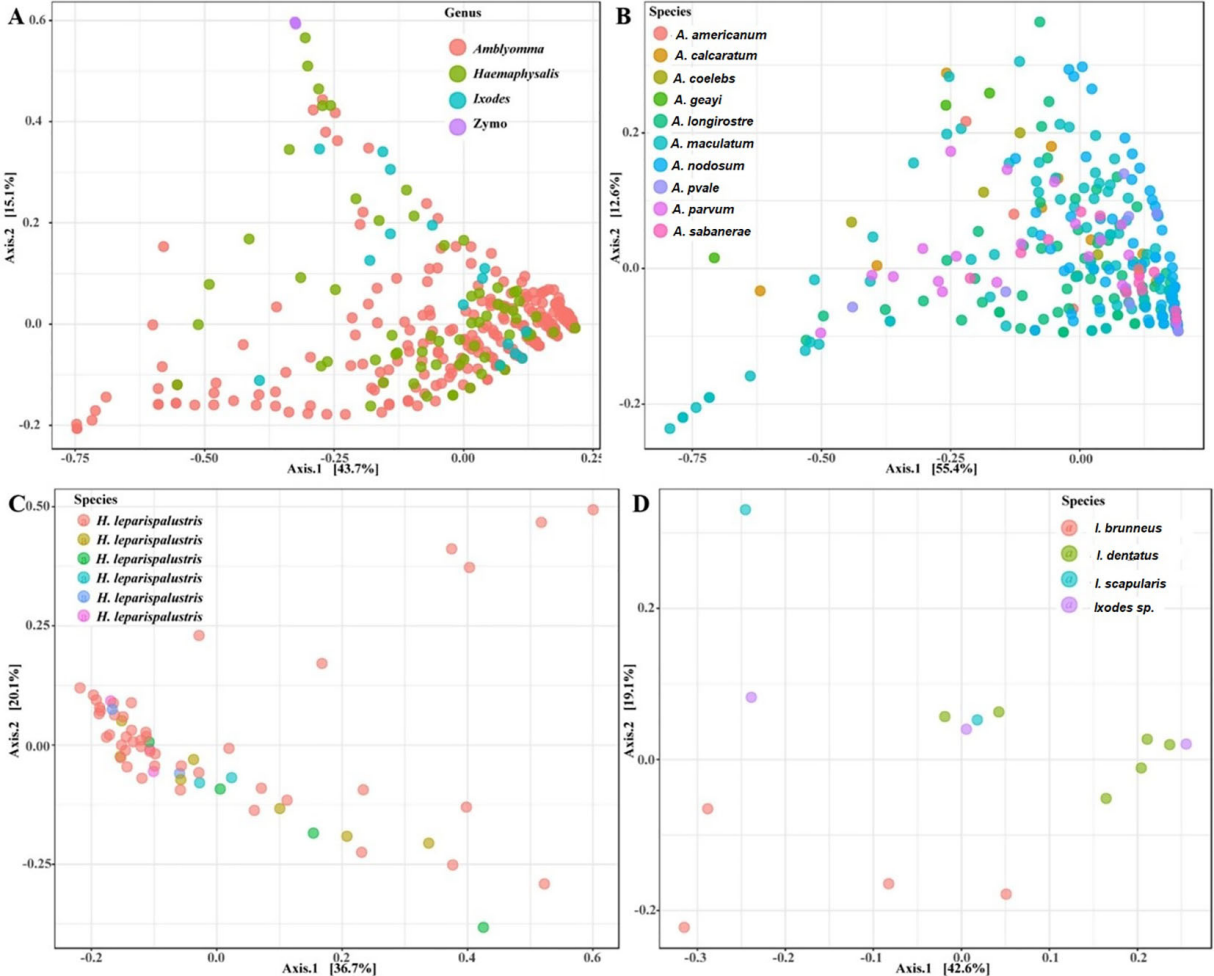
Principal coordinate analysis of beta diversity measures for **(A)** all tick genus, **(B)** *Amblyomma* **(C)** *Ixodes* and **(D)** *Haemaphysalis leporispalustris* ticks using the Bray-Curtis similarity metrics. Highlighted ellipses indicate measures of confidence based on degree of variations. Zymo represent the DNA from the mock bacterial communities.

Our analysis of microbial communities revealed significant differences in the bacterial communities between all tick genera (*Amblyomma, Ixodes*, and *Haemaphysalis*). The NMDS analysis of community structure using the Bray-Curtis distance matrix (PERMANOVA: F = 30.04, R^2^ = 0.23, P < 0.001, [NMDS] Stress = 0.11) revealed overlapping clusters across all tick genera (**Figure 4A**; **Supplementary Figure S4A**). Several *Amblyomma* and *Haemaphysalis* ticks were seen to cluster separately on the lower axis of the PCoA (**Figure 4A**).

Our observations indicated that tick species exhibiting close clustering on the PCoA plot demonstrated similar abundance levels for both *Francisella* and *Rickettsia* (**Supplementary Figures S4B, C**). While most of the *Amblyomma* species tend to share similar microbial communities based on the PERMANOVA (F = 10.33, R^2^ = 0.24, P < 0.001), several *Amblyomma* ticks, in particular *A. maculatum, A. calcaratum, A. geayi*, and *A. longirostre*, were outliers, indicating significantly different community structure among *Amblyomma* species (**Figure 4B**). The observed clustering patterns in *Amblyomma* ticks, including the outlier clustering of certain *Amblyomma* species, can be attributed to variations in the abundance of *Rickettsia* and *Francisella* (**Supplementary Figures S5A, B**). *Ixodes* ticks differed significantly according to the PERMANOVA of the Bray-Curtis distances (F = 2.60, R^2^: 0.41, P < 0.004). Unlike the *Amblyomma* and *Ixodes* ticks, only one *Haemaphysalis* species was part of this study, and no unique clustering was observed in this species. Similarly, the PERMANOVA test of the Bray-Curtis distances was not statistically significant (F = 1.2002, R^2^: 0.09525, p-value: 0.228) (**Figure 4C**). Distinct clustering was observed with *I. dentatus, I. scapularis* and *I. sp*, while *I. brunneus* clustered separately as explained by 18.2% (Axis 1) and 44.8% (Axis 2) of the PCoA (**Figure 4D**; **Supplementary Figures S5C, D**).

### Tick microbial interactions

**Figure 5:**
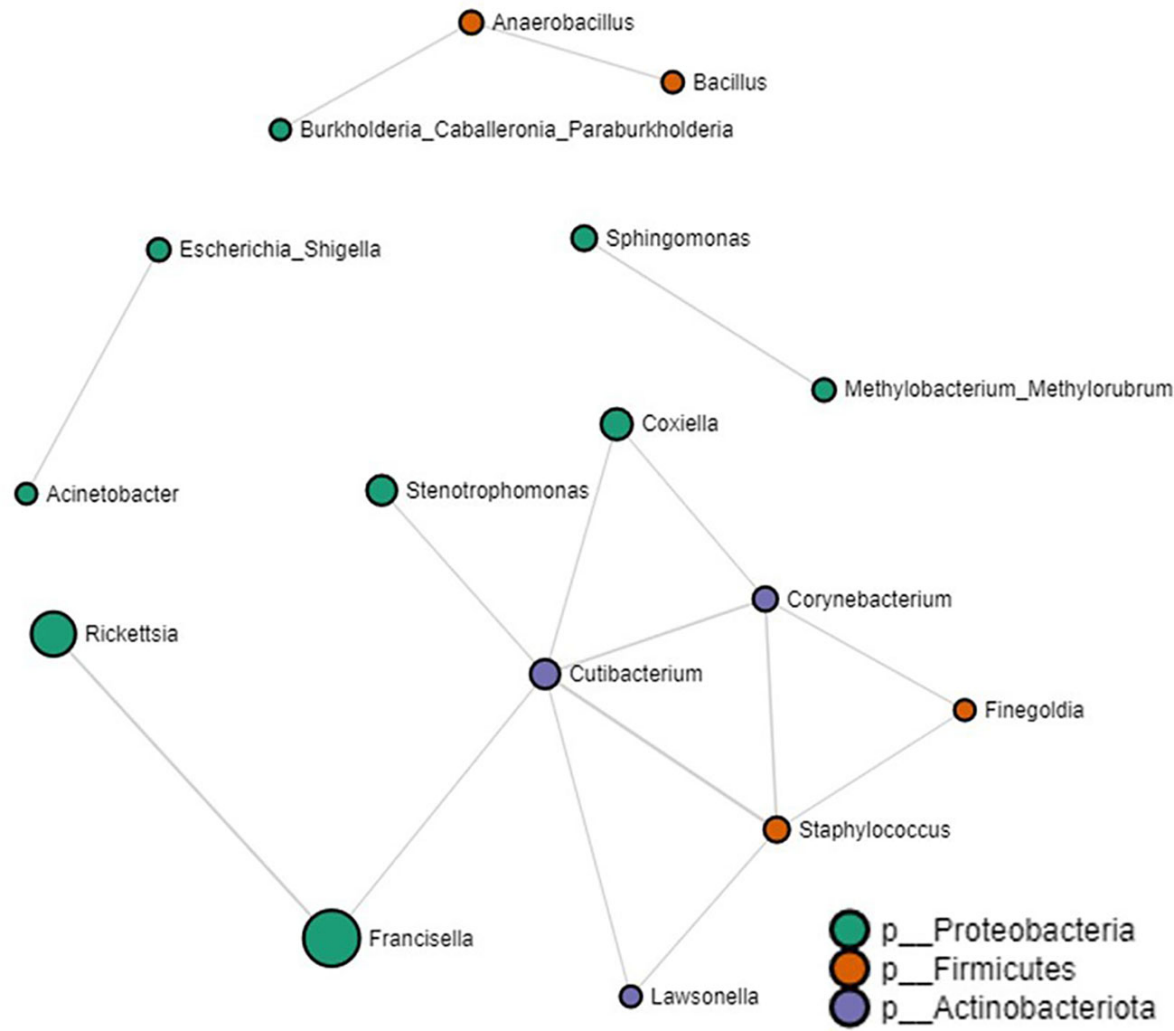
Correlation network analysis on all tick genera. Correlation network generated using the SparCC algorithm. Correlation network with nodes representing taxa at the family level and edges representing correlations between taxa pairs. Node size correlates with the number of interactions in which a taxon is involved. The color-coded legend shows the bacterial phyla.

Our network analysis identified 16 significant partial correlations between 12 OTUs. 87% of interactions were identified as positive correlations (**Supplementary Table S4**). We identified negative partial correlations between the prevalence of the *Francisella* genus and both *Rickettsia* and *Cutibacterium*. Notably, these were the exclusive bacteria that showed interactions with *Francisella*. Within the network interactions, four unique clusters were identified with the largest clusters consisting of bacteria commonly reported within the tick microbiome such as *Coxiella, Francisella, Rickettsia, Staphylococcus* and *Cutibacterium* (**Figure 5**. Log-transformed counts of reads assigned to *Rickettsia* (p-value: 0.001022, FDR: 0.012008)*, Francisella* (p-value: 3.7672e-11, FDR: 5.3117e-9), and *Coxiella* (p-value: 0.005503, FDR: 0.045643) showed significantly higher abundance in *Amblyomma* ticks (**Supplementary Figure S6**). At the genus level, 65 microbe-microbe interactions were observed in the *Amblyomma* network, 54 in the *Ixodes* network, and 48 in the *Haemaphysalis* network. Detailed microbe-microbe interactions observed amongst each tick genus can be found in the additional file section (**Supplementary Tables S5**-**S9**).

### Species-specific microbial interactions: Amblyomma

Sixty-two percent of the total microbial interactions from the *Amblyomma* dataset were identified as positively correlated (**Supplementary Table S5**). Due to the differences in the numbers from each *Amblyomma* species, they were divided into samples from a large sample size (> 20) and a small sample size (< 20). In the network analysis performed on the datasets with large sample sizes (*A. calcaratum, A. longirostre, A. maculatum, A. nodosum, A. parvum*, and *A. sabanerae*), we observed 56 positive and 46 negative significant partial correlations (**Supplementary Figure S7**; **Supplementary Table S6**). Several of the identified genera belonged to environmental groups and a few bacteria were identified in other arthropod microbiomes such as *Wolbachia* and *Spiroplasma*. *Spiroplasma* was positively correlated with both *Wolbachia* and *Coxiella*. However, we did observe negative partial correlations between *Francisella* and *Rickettsia* similar to the whole tick dataset. *Candidatus Midichloria* was positively correlated with *Methylobacterium Methylorubrum* and *Escherichia Shigella*, which were both from environmental sources (**Supplementary Figure S7**). Of the datasets with small sample sizes (*A. americanum, A. coelebs, A. geayi, A. ovale, A. triste*, and *A. varium*), 25 microbial interactions were positive significant correlations, while 10 were negatively correlated (**Supplementary Figure S8**; **Supplementary Table S7**). From the network analysis, 5 unique clusters were identified, with the largest involving interactions between 10 bacteria genera. In contrast to the *Amblyomma* datasets with large representation, *Francisella* was not detected within any of the network clusters. In addition, *Coxiella* was positively correlated with *Sphingomonas*, while *Candidatus Midichloria* was negatively correlated with *Bacillus*.

### Species-specific microbial interactions: Ixodes

Of the 54 significant partial interactions detected in the dataset from *Ixodes* ticks, 42 were positive interactions, while 12 were negative interactions (**Supplementary Table S8**). From the network analysis, three different clusters were identified. However, we did not detect *Francisella* and *Rickettsia* in the *Ixodes* network dataset, indicating that other microbes, which include several of the bacteria identified as environmental groups, are more important in driving the microbial interactions. We observed positive partial correlations between *Anaerobacillus, Wolbachia* and *Bacillus* and between *Wolbachia* and *Microbacterium* (**Supplementary Figure S9**; **Supplementary Table S8**). *Candidatus Midichloria* only interacted with *Lawsonella* and both show partial positive correlations.

### Species-specific microbial interactions: Haemaphysalis

In the network analysis performed on *Haemaphysalis* ticks, we observed 48 significant partial positive correlations and 10 significant partial negative correlations, which were grouped into three unique network clusters (**Supplementary Figure S10**; **Supplementary Table S9**). Two of the clusters involved commonly reported environmental group bacteria with previous associations to ticks and arthropods and were linked through direct or indirect interactions with *Rickettsia* (**Supplementary Figure S10**). *Rickettsia* was positively correlated to *Spiroplasma* and *Candidatus* Midichloria but negatively correlated with *Massilia*. *Candidatus Midichloria* was positively correlated to *Stenotrophomonas*, while *Stenotrophomonas* was also positively correlated to *Coxiella* (**Supplementary Figure S10**, **Supplementary Table S9**).

### Spatial analysis of parasitized birds

**Figure 6:**
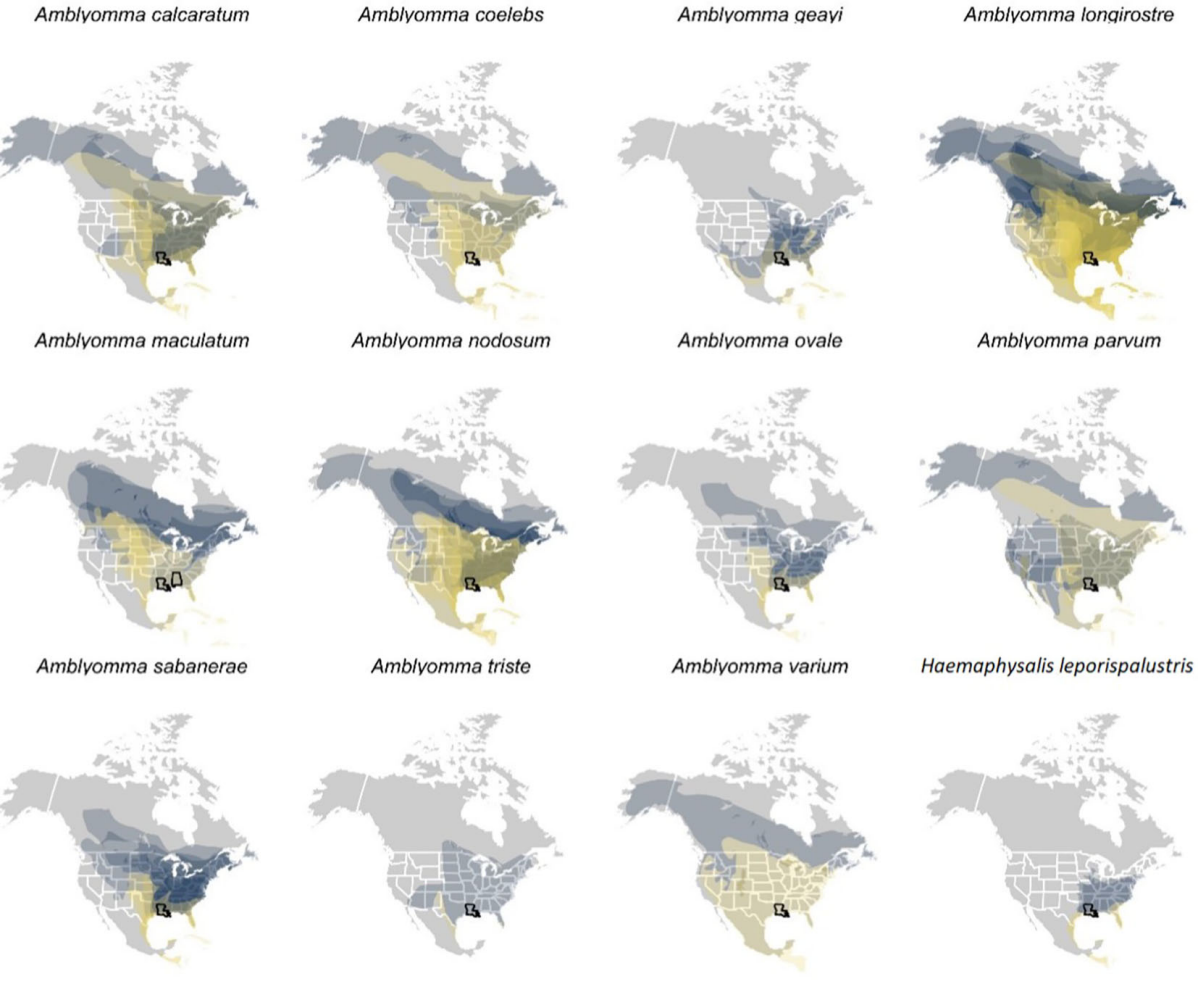
International Union for Conservation of Nature (IUCN) distributions of those bird species and prediction of their potential spread. Each plot is for a different tick species, so some ticks are obviously found on more species than others. The IUCN distributions includes the breeding range (blue) and the migration range (yellow), a prediction of where the migratory birds could spread ticks during stopover (yellow) or upon their arrival to the breeding area (blue).

**Figure 7:**
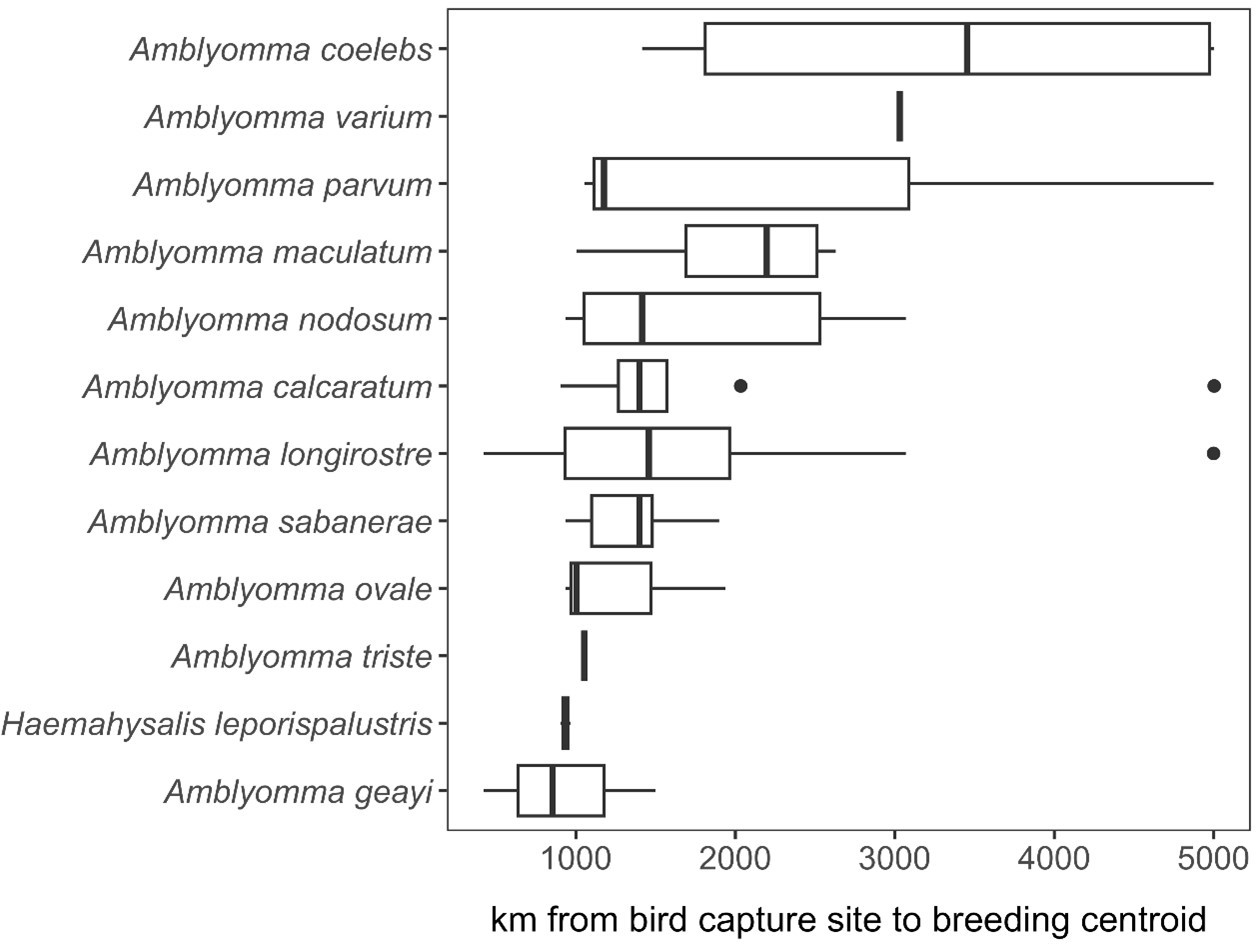
Estimated dispersal distance of each tick species recovered from migratory birds in spring.

A total of 17,550 birds (comprising 14,929 uniquely banded individuals) were captured and sampled for ticks, encompassing 101 bird species. 1,721 individual birds were recaptured at least once. Among our entire sample, 164 individual birds, representing 28 bird species, were found to have ticks. Of the 28 bird species, we focused our spatial analyses on the 21 species of migratory birds sampled in spring for which ticks could be molecularly identified to species. Geographies of plausible tick and pathogen dispersal varied widely among the 12 tick species (**Figure 6**). This analysis suggested migratory birds using the Gulf of Mexico during spring migration could disperse *A. calcaratum*, *A. coelebs*, *A. longirostre*, *A. maculatum*, *A. nodosum*, *A. parvum, A. sabanerae*, *and A. varium* possibly as far as the Northwest Territories and Alaska, whereas *A. geayi*, *A. ovale*, *A. triste*, and *H. leporispalustris* had overall less dispersal capacity (e.g., north into the Great Lakes region) owing to the shorter-distance migrations of their avian hosts. Formal analysis of distances between spring migration capture sites and breeding range centroids for parasitized birds (n = 69 unique combinations of bird species, tick species, and sites) demonstrated dispersal capacity ranged from 421 to 5003 kilometers (**Figure 7**). On average, *A. geayi* had the least dispersal capacity (x̄ = 925 km), whereas *A. coelebs* had the most extreme dispersal capacity (x̄ = 3,331 km). A GLM excluding the two tick species with only one parasitized bird species or capture site (*A. triste* and *A. varium*) suggested such dispersal capacity among tick species to be non-significant (χ^2^ = 15.52, P = 0.08).

## Discussion

This study aimed to assess the role of birds in introducing exotic neotropical tick species from Central and South America into North America. In addition, we also aimed to investigate whether these ticks could serve as indicators for monitoring the introduction of both known and previously unidentified pathogenic bacteria into new geographical regions. Metagenomic sequencing also revealed significant diversity in the microbial communities within the ticks, members of which include bacteria in the pathogenic and symbiotic genera. During our study, the highest abundance of ticks was observed in late spring, particularly in April, where over 65% of the total ticks were collected. It is important to highlight that tick collection spanned across the entire spring and autumn seasons, indicating a temporal distribution of ticks beyond the peak observed in late spring. In the current study, we aimed to characterize the microbiome of ticks collected from birds migrating to and from the USA, since they can disperse tick species and their associated pathogens. Our previous work during spring migration in Louisiana identified Neotropical tick species including *Haemaphysalis juxtakochi*, *Amblyomma longirostre*, *A. nodosum*, *A. calcaratum*, *A. maculatum*, and *H. leporispalustris* (Budachetri et al., 2017). The identified ticks were exotic species that originated outside of the USA (Budachetri et al., 2017). Our 12S rRNA analysis here identified three tick genera—*Amblyomma, Ixodes*, and *Haemaphysalis*—based on the homology search. These findings further support the role of migratory birds in the introduction and dispersal of ticks in the general *Haemaphysalis* and *Amblyomma* as previously reported (Budachetri et al., 2017).

The bacterial composition within each tick species revealed a predominant presence of the endosymbiont *Francisella*, representing over 50% of the detected bacterial abundance. Subsequently, *Rickettsia* was identified as the second most abundant bacterial species. In *A. varium*, only the genus *Francisella* was identified within the bacterial composition. In various sampling locations and with a diverse range of bird hosts, the consistent presence of *Francisella* and *Rickettsia* in these tick microbiomes underscores their pivotal roles as core members. This association suggests a potential symbiotic reliance of these tick species on these microbial entities, possibly for nutritional or reproductive dependence (Gerhart et al., 2016). These genera of endosymbionts are all members of the phylum Proteobacteria which comprises the majority of the bacterial species detected in hard ticks (Narasimhan et al., 2007). Members of these groups have primarily been documented in adult ticks collected from the field or maintained in laboratory conditions, often with the presence of only one or two endosymbionts (Narasimhan et al., 2007). Contrary to this pattern, our study indicates that while the majority of tick species harbor primarily *Francisella* and *Rickettsia*, our observations reveal specific tick species with additional components in their core microbiome. Notably, alongside *Francisella* and *Rickettsia*, *Candidatus* Midichloria and *Coxiella* consistently coexisted within the core microbiome of *A. calcaratum*, *A. coelebs*, *A. geayi*, *A. maculatum*, and *I. brunneus*. Both *Candidatus* Midichloria and *Francisella*-like endosymbionts have been most commonly reported in ixodid ticks (Budachetri et al., 2018; Duron et al., 2018; Rio et al., 2016; Adegoke et al., 2022; Gofton et al., 2015; Sassera et al., 2008). There are similarities in the bacteria identified from ticks in this study and those described from our previous. *Candidatus* Midichloria*, Rickettsia* and *Francisella* were amongst the dominant genera detected in ticks collected off migratory passerine birds from the neotropics moving into the United States (Budachetri et al., 2017).

The vertical maintenance of these endosymbionts would explain their co-existence in these tick species at lower developmental stages and a gradual decline as they molt into adult ticks (Menchaca et al., 2013). It has been reported that the presence of specific endosymbiont genera in earlier developmental stages does not necessarily result in the transmission of these bacteria to the subsequent stage. Noda et al. reported that only 50% of *Rickettsia* positive *I. scapularis* nymphs remained positive after molting to adult (Noda et al., 1997). One of the studies has indicated reduction in the relative abundance of the *Coxiella* endosymbiont by more than half in the adult compared to the nymphal stages of the *A. americanum* tick (Menchaca et al., 2013). However, if the *Coxiella* endosymbiont decreases throughout its growth, it brings into question how this bacterium is transmitted from females to their eggs. If this decline is due to the effects of feeding followed by starvation over time, or if aging itself is a factor. Additional work is needed to identify the factors contributing to this decline.

*Spiroplasma*, an endosymbiont well reported in dipteran vectors (Karatepe et al., 2018; Son et al., 2021) was also present in *Haemaphysalis* ticks in the current study. *Spiroplasma* was detected as a member of the *H. leporispalustris* microbiome, which could suggest an endosymbiotic relationship. While *Spiroplasma* species are well known for their male-killing attribute, several species of this symbiont do not induce male killing and present different phenotypes and genetic makeup, several of which benefit the host (Arai et al., 2022).

While we observed no significant variations in microbial diversity among tick genera, *Amblyomma* exhibited the lowest alpha diversity as determined by OTU abundance and the Shannon index. This reduced diversity appears to be influenced by the notably high prevalence of *Francisella*, however, confirming this relationship would require further experimental validation. The variations in alpha diversity among the biological replicates of the mock controls may be attributed to the bacterial composition of the mock DNA. While all the Operational Taxonomic Units (OTUs) detected in the mock communities were anticipated, the differing composition between each mock replicate could be responsible for the high degree of variability observed in alpha diversity. By applying PCoA analysis, we were able to visualize clustering in the microbial structure of tick genera, which illustrated several similarities and differences. Several tick species and genera share similar microbial profiles based on OTU abundance. Our findings also show that differences in the abundances of *Francisella* and most importantly *Rickettsia*, the two most abundant bacteria, were significant in shaping the PCoA cluster patterns. Our results show that ticks with high abundance of either *Francisella* or *Rickettsia* cluster separately from each other. Several reports have detected *Rickettsia* in exotic ticks collected from birds migrating into North America, especially the USA (Miller et al., 2016; Souza et al., 2018; Budachetri et al., 2017) and Canada (Morshed et al., 2005; Ogden et al., 2015). However, the prevalence of these *Rickettsia* in ticks within the USA and the ability of these ticks to serve as competent vectors of *Rickettsia* remains poorly understood.

The observed microbial interactions within different tick genera, as revealed by network analysis, provide valuable insights into the dynamics of the tick microbiome and its potential functional implications. The predominance of positive interactions, especially when considering the entire tick dataset, suggests a prevalent of cooperative relationship among the microbial entities associated with ticks for which several factors such as co-feeding, season which ticks were collected, multiple host and even geographical range could have influenced (Lalzar et al., 2012; Williams-Newkirk et al., 2014).

The absence of pathogenic genera within the network of *Ixodes* ticks is noteworthy and may indicate a distinct microbial composition or a unique ecological niche in this tick genus. This finding contrasts with the networks of other tick genera, where the presence of environmental bacteria alongside tick endosymbionts and pathogens suggests a more complex and interconnected microbial community. The coexistence of *Rickettsia*, a known pathogenic genus, with various tick and non-tick symbionts in the *Haemaphysalis* dataset raises intriguing questions about the potential roles of these bacteria in shaping microbes-microbes interactions or even microbe-tick interactions. Ticks are known to harbor both pathogenic and symbiotic *Rickettsia* (Burgdorfer et al., 1981) and the presence of genes required for vitamin B synthesis in the genomes of some of these *Rickettsia* indicate they provide nutritional support to the tick host (Hunter et al., 2015). Similarly, different commensal and endosymbiont reportedly alter host feeding behavior as seen in ticks (Zhong et al., 2021) and dipterans (Leitão-Gonçalves et al., 2017). Reports have also shown that, in ticks, *Rickettsia* endosymbionts influences motility in *A. americanum, Dermacentor variabilis* and *I. scapularis* larva (Kagemann and Clay, 2013). The significant positive correlation between *Rickettsia*, *Wolbachia*, and *Spiroplasma* hints at a potential synergistic or mutually beneficial relationship among these bacteria genera, adding a layer of complexity to our understanding of microbial interactions within ticks. As these birds cover thousands of kilometers with minimal stops in between, the heightened energy expenditure and mechanical demands on immature ticks imply a reliance on endosymbionts, such as *Rickettsia*, for both nutritional support and behavioral adaptations.

Overall, these observations underscore the need for a nuanced perspective on the tick microbiome, acknowledging the diversity of interactions and potential functional roles played by different bacteria. The findings contribute to our understanding of the intricate relationships within tick-associated microbial communities, offering insights that may have implications for tick-borne diseases and ecological dynamics.

The findings from the current study support the hypothesis that migratory birds contribute to the seasonal introduction of non-native tick species and their associated microbes. Our analysis of the distribution of parasitized migratory birds suggests spring migration could facilitate dispersal hundreds to thousands of kilometers into the USA, with exotic ticks such as *Amblyomma coelebs, A. varium, A. parvum, A. nodosum, A. calcaratum* and several other ticks within this genus plausibly able to spread particularly far from where they were captured and sampled at stopover sites along the northern Gulf of Mexico.

## Conclusion

This study highlights the role migratory birds can play in introducing non-native tick species and their associated microbes into the USA. Our results demonstrate that songbirds have the ability to introduce exotic tick species during their seasonal migration into North America, with 421 ticks collected from 28 songbird species. Irrespective of the tick species, the core bacterial genera identified include *Francisella, Rickettsia, Spiroplasma*, and *Candidatus Midichloria*. The study provides valuable insight into the mechanisms of tick dispersal, emphasizing the importance of understanding bird migration patterns in predicting the introduction and establishment of potentially invasive tick populations. These findings contribute to the knowledge of the ecological factors influencing the spread of ticks and their associated pathogens, informing future strategies for surveillance and control efforts.

## Data Availability

The datasets presented in this study can be found in online repositories. The names of the repository/repositories and accession number(s) can be found in the article/**Supplementary Material**.
