# Supplementary material for: Ticks without borders: microbiome of immature neotropical tick species parasitizing migratory songbirds along northern Gulf of Mexico
Source: Front Cell Infect Microbiol. 2024 Nov 18;14:1472598. doi: 10.3389/fcimb.2024.1472598 (PMC11609183; doi:10.3389/fcimb.2024.1472598)
Supplement: Supplementary file 1 [file DataSheet1.docx]

SUPPLEMENTARY Figures


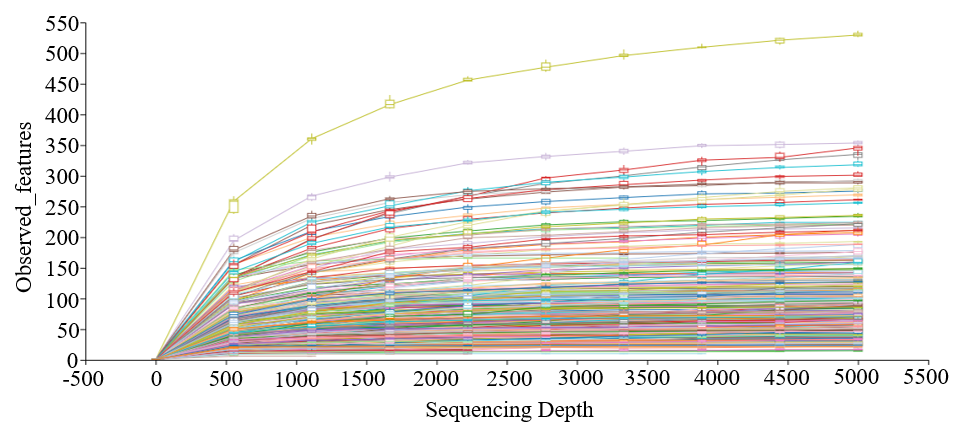


Figure S1: Rarefaction analysis of raw reads from all sampled ticks. Each curve represents an individual tick, and all tick sequences were normalized to a sequence depth of 5000.


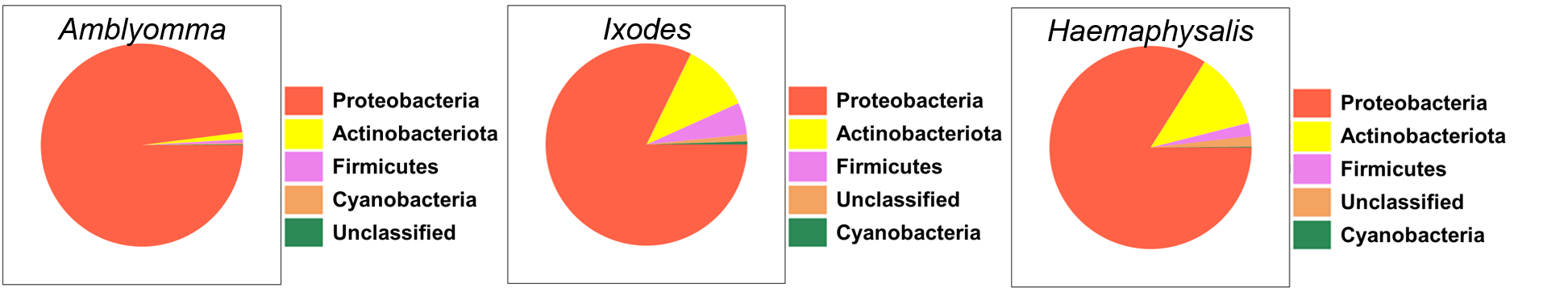

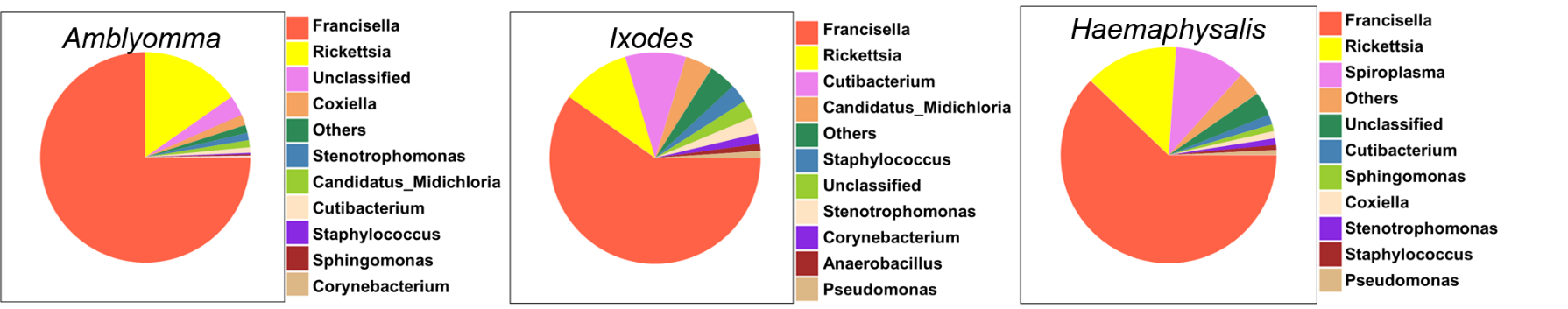


**A**

**B**

Figure S2: Pie chart summary of read abundance at A) phylum and B) genus level showing the taxa represented at an abundance of more than 1%.


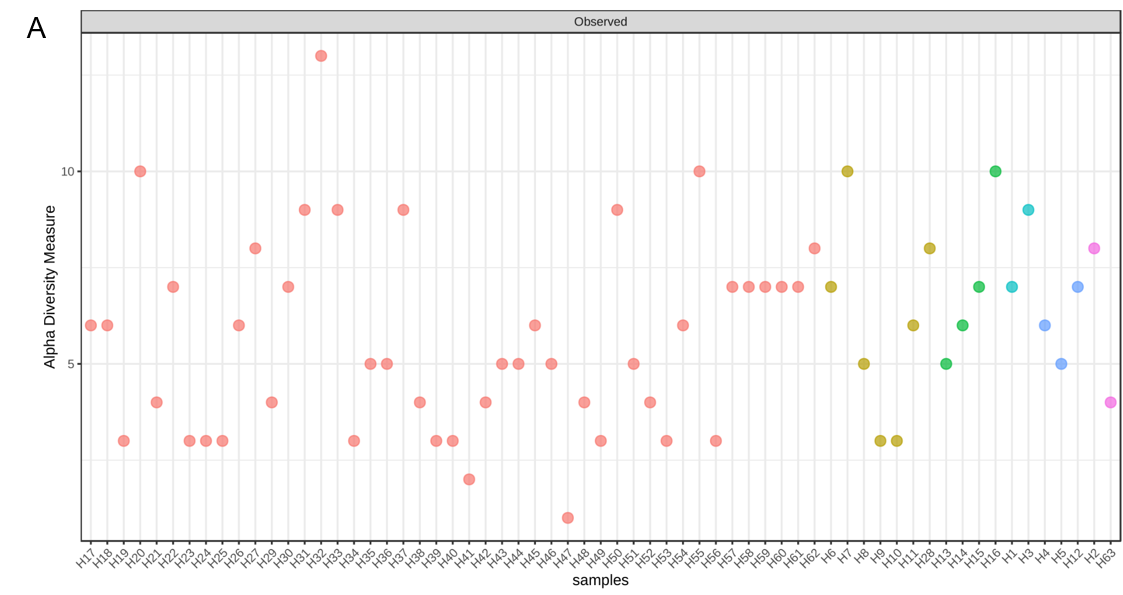

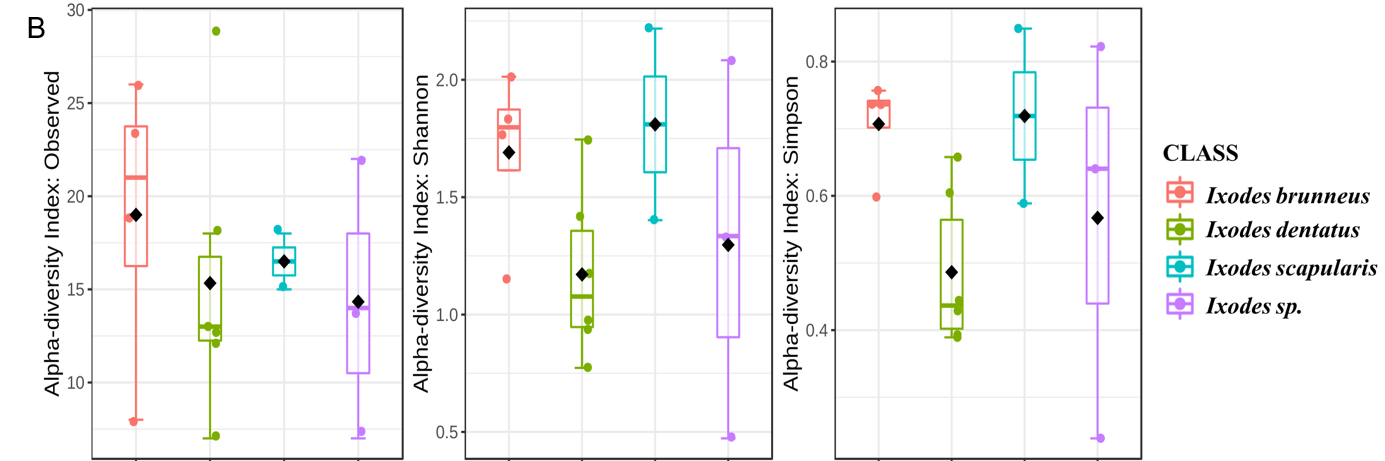


Figure S3: Alpha-diversity analysis of A) *Haemaphysalis leporispalustris* (p-value: 0.57981; [Kruskal-Wallis] statistic: 3.7915) showing individual tick replicates and B) *Ixodes* ticks showing combined result. Alpha diversity for *Ixodes* was analyzed using the Observed OTUs (p-value: 0.57037; [Kruskal-Wallis] statistic: 2.0098), Shannon’s Index (p-value: 0.28437; [Kruskal-Wallis] statistic: 3.7958) and Simpson’s Index metrics (p-value: 0.23367; [Kruskal-Wallis] statistic: 4.2708).


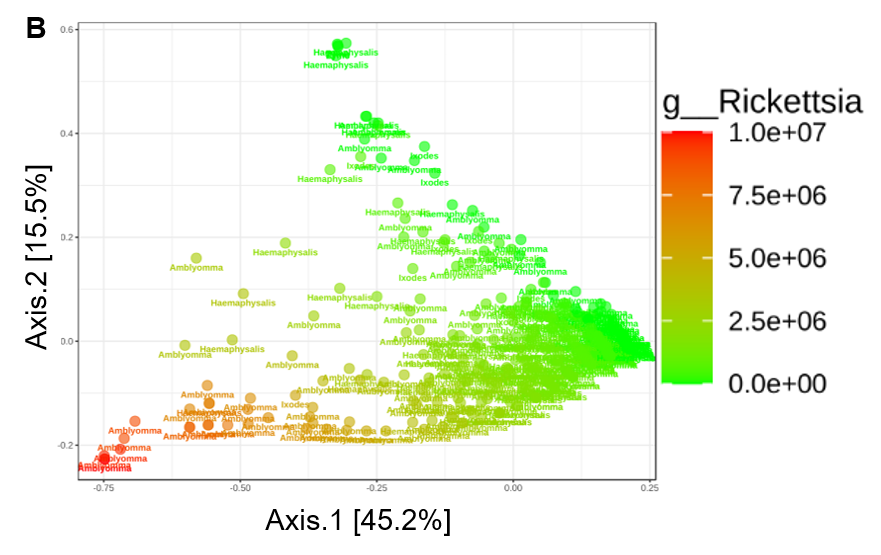

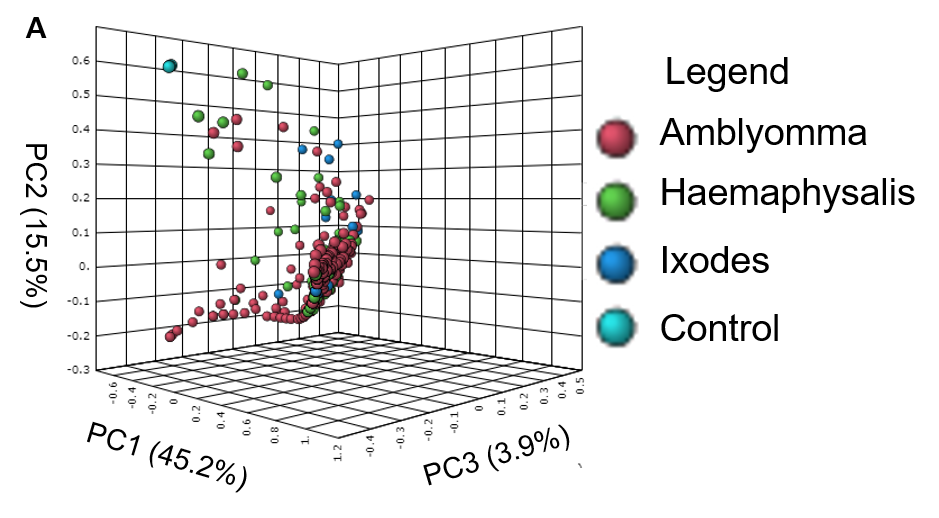


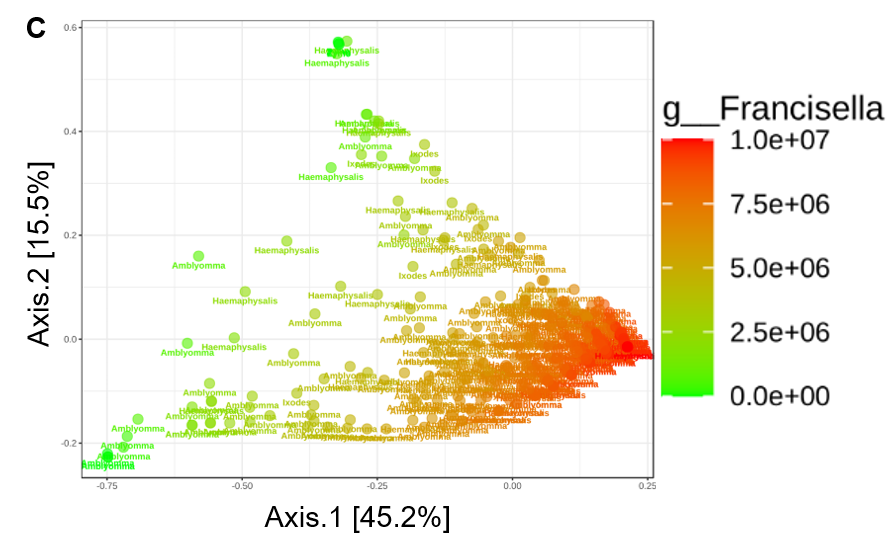


Figure S4: Principal coordinate analysis of beta-diversity measures for all tick datasets using the Bray-Curtis distance measures showing A) 3-D rendering of the PCoA. PCoA analysis based on read abundance showing the distribution of B) *Rickettsia* and C) *Francisella* and how the two genera shape the beta diversity.


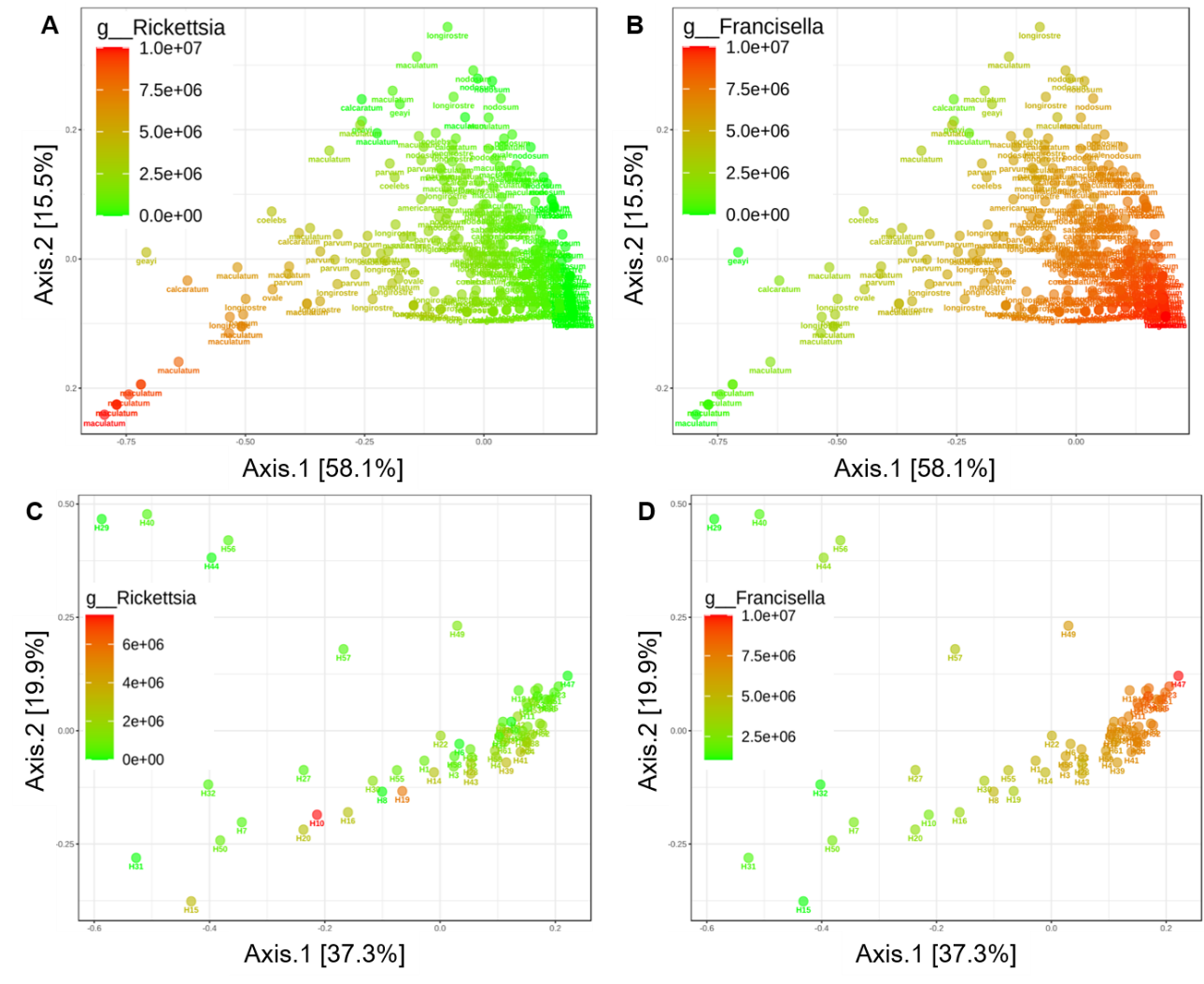


Figure S5: PCoA and read abundance distribution of *Rickettsia* and *Francisella* in A, B) *Amblyomma* and C, D) *Ixodes* ticks.


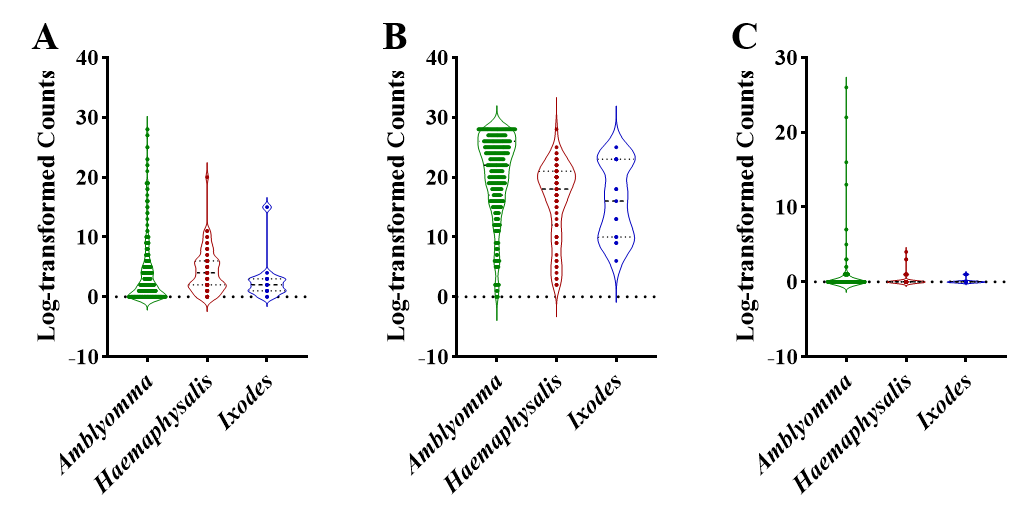


Figure S6: Violin plots of Log-transformed counts of reads assigned to A) *Rickettsia,* B) *Francisella,* and C) *Coxiella* among *Amblyomma, Haemaphysalis,* and *Ixodes* ticks.


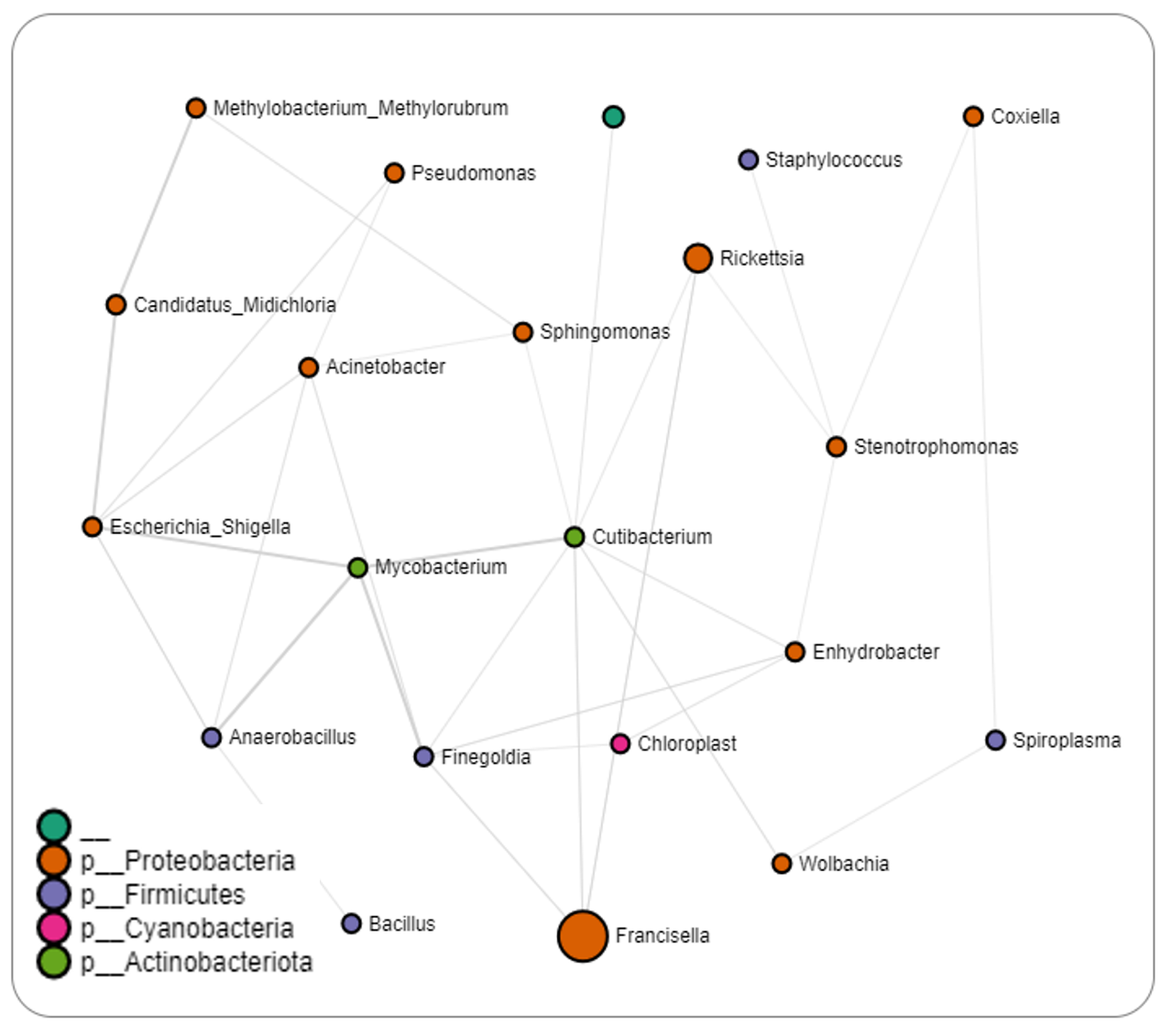


Figure S7: Correlation network analysis on *A. calcaratum, A. longirostre, A. maculatum, A. nodosum, A. parvum,* and *A. sabanerae*. The correlation network generated using the SparCC algorithm. Correlation network with nodes representing taxa at the family level and edges representing correlations between taxa pairs. Node size correlates with the number of interactions in which a taxon is involved. The color-coded legend shows bacterial phyla. Nodes without a label represents unidentified taxa.


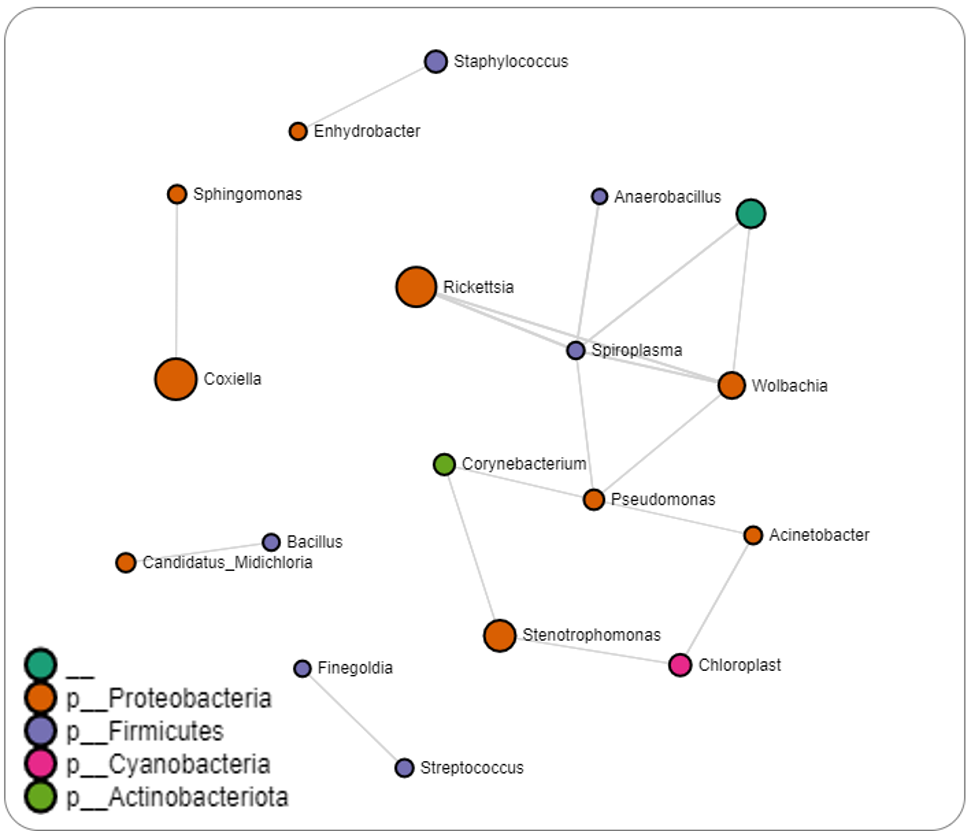


Figure S8: Correlation network analysis on *A. americanum, A. coelebs, A. geayi, A. ovale, A. triste,* and *A. varium*. The correlation network is generated using the SparCC algorithm. Correlation network with nodes representing taxa at the family level and edges representing correlations between taxa pairs. Node size correlates with the number of interactions a taxon involves. The color-coded legend shows bacterial phyla.


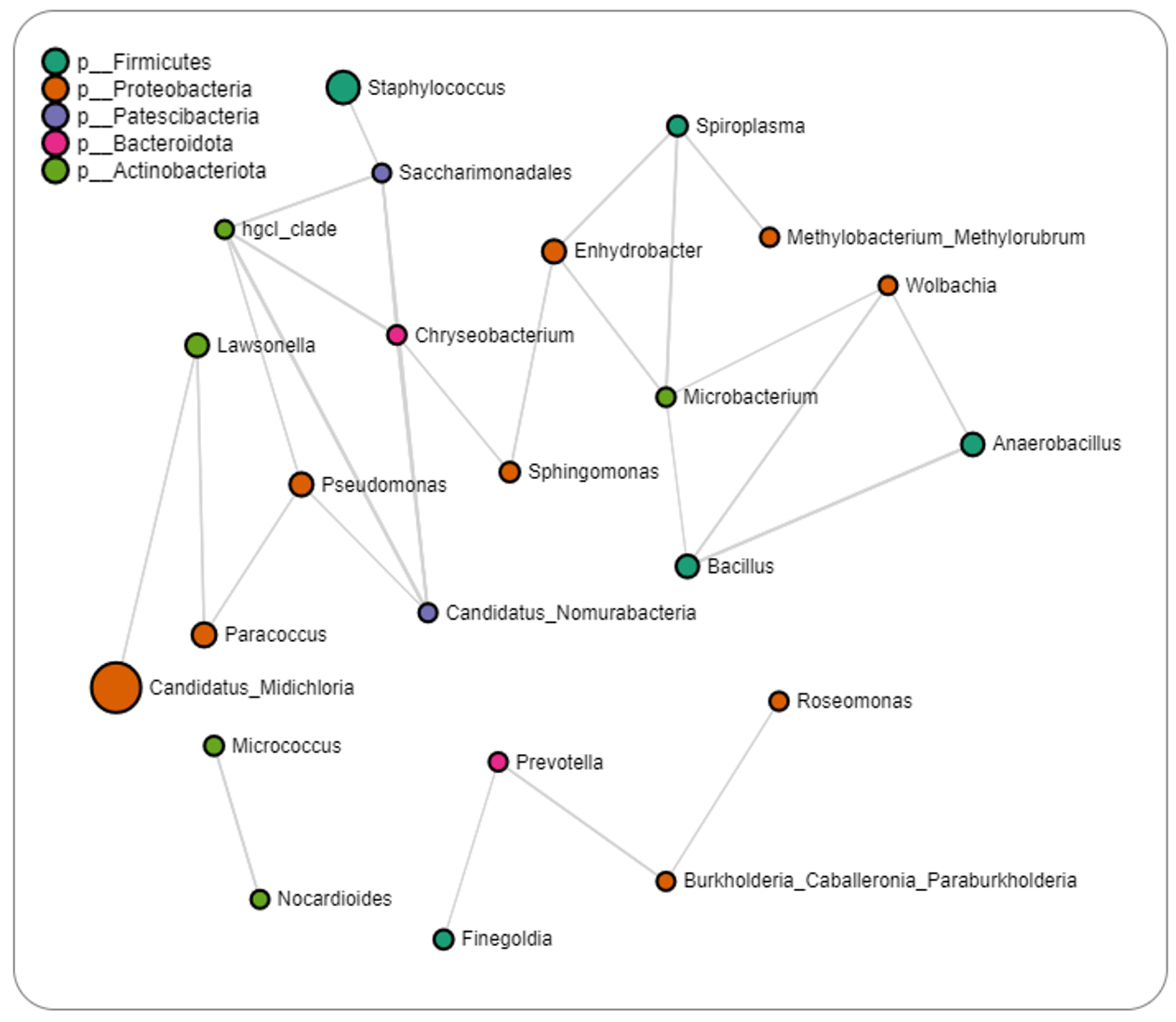


Figure S9: Correlation network analysis on *Ixodes* ticks. The correlation network generated using the SparCC algorithm. Correlation network with nodes representing taxa at the family level and edges representing correlations between taxa pairs. Node size correlates with the number of interactions in which a taxon is involved. The color-coded legend shows the bacterial phyla. Nodes without a label represents unidentified taxa.


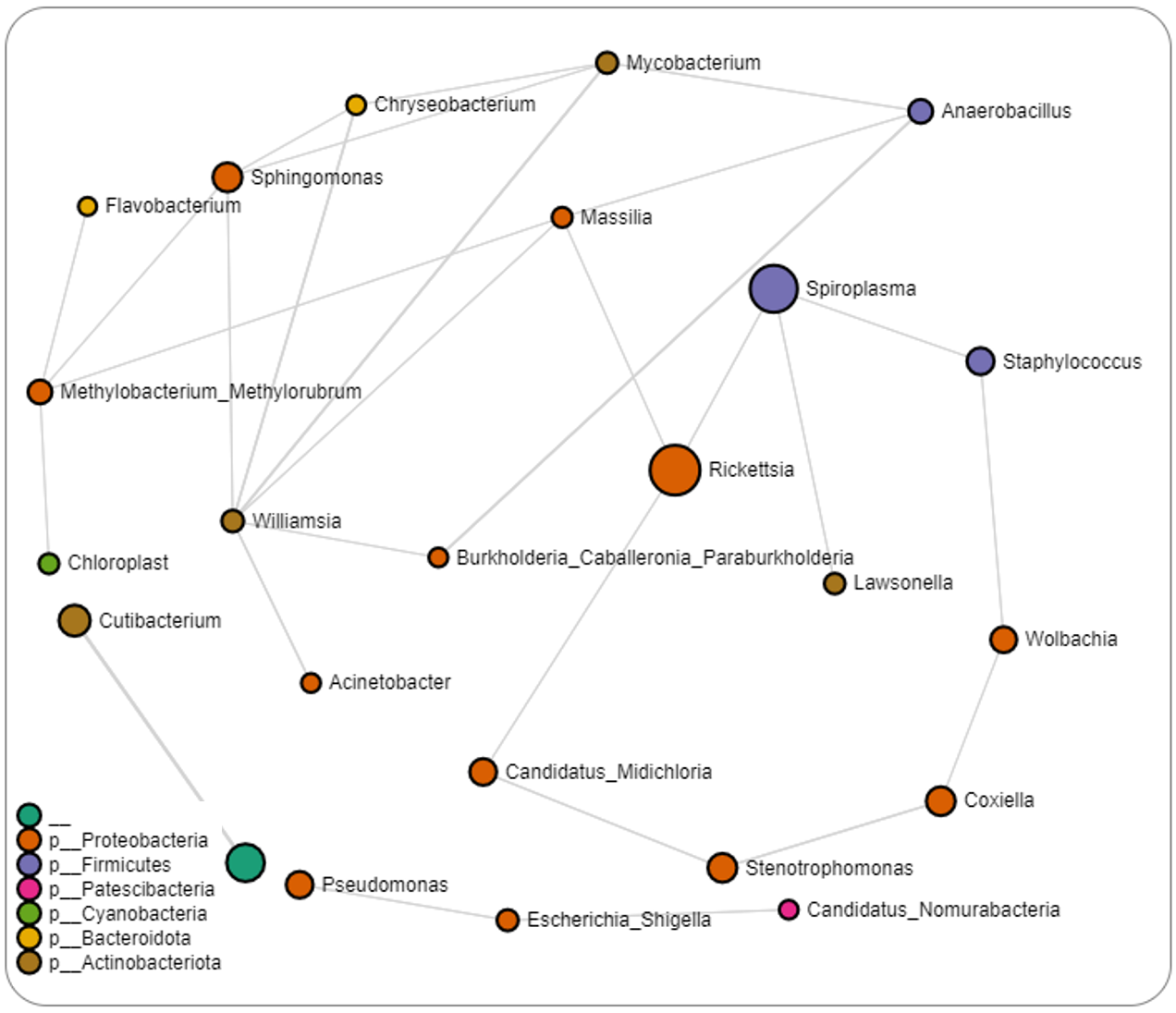


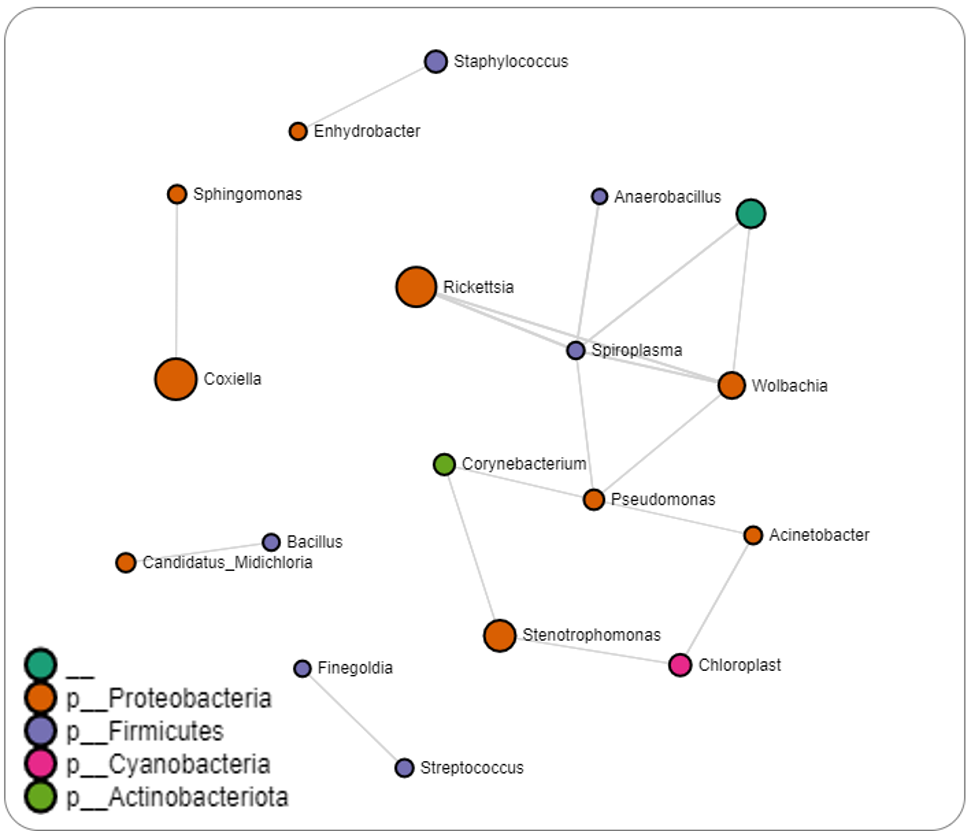


Figure S10: Correlation network analysis on *Haemaphysalis leporispalustris*. Correlation network generated using the SparCC algorithm. Correlation network with nodes representing taxa at the family level and edges representing correlations between taxa pairs. Node size correlates with the number of interactions in which a taxon is involved. The color-coded legend shows the bacterial phyla.
